# Molecular Mechanisms of Tumor Immunomodulation in the Microenvironment of Colorectal Cancer

**DOI:** 10.3390/ijms23052782

**Published:** 2022-03-03

**Authors:** Dorothea Plundrich, Sophia Chikhladze, Stefan Fichtner-Feigl, Reinhild Feuerstein, Priscilla S. Briquez

**Affiliations:** 1 Department of General and Visceral Surgery, Medical Center—University of Freiburg, Faculty of Medicine, University of Freiburg, 79106 Freiburg, Germany; dorothea.plundrich@uniklinik-freiburg.de (D.P.); sophia.chikhladze@uniklinik-freiburg.de (S.C.); stefan.fichtner@uniklinik-freiburg.de (S.F.-F.); reinhild.feuerstein@uniklinik-freiburg.de (R.F.); 2 Department of Biomedical Sciences, Cedars-Sinai Cancer Institute, Cedars-Sinai Medical Center, 8700 Beverly Blvd, Los Angeles, CA 900048, USA; 3 Department of Medicine, Cedars-Sinai Cancer Institute, Cedars-Sinai Medical Center, 8700 Beverly Blvd, Los Angeles, CA 900048, USA

**Keywords:** colorectal cancer, molecular interactions, tumor microenvironment, immune responses, cell-cell interactions, cytokines, extracellular matrix, microbiome

## Abstract

Colorectal cancer remains one of the most important health challenges in our society. The development of cancer immunotherapies has fostered the need to better understand the anti-tumor immune mechanisms at play in the tumor microenvironment and the strategies by which the tumor escapes them. In this review, we provide an overview of the molecular interactions that regulate tumor inflammation. We particularly discuss immunomodulatory cell-cell interactions, cell-soluble factor interactions, cell-extracellular matrix interactions and cell-microbiome interactions. While doing so, we highlight relevant examples of tumor immunomodulation in colorectal cancer.

## 1. Introduction

Colorectal cancer is the second leading cause of cancer-related deaths in the world, accounting for nearly 1 million deaths in 2020. In addition, colorectal cancer generates a high economic burden, estimated to be around €19.1 billions in Europe in 2015 [1]. Whereas the majority of primary colorectal cancers can be eradicated through surgical resection, only a minority of patients diagnosed with metastatic CRC (mCRC) can be cured by surgery. Therefore, mCRC patients have to undergo additional or alternative treatments, such as chemotherapy, monoclonal antibody treatment (e.g, Cetuximab, Panitumumab), immunotherapy, radiofrequency ablation, or a combination of these [2,3]. Despite all efforts, the prognosis for patients with mCRC remains poor, with a 5-year survival rate lower than 20% [4]. Therefore, there is an urge to improve the current therapies for mCRC.

The emergence of cancer immunotherapies in the last decade has provided new hopes for the treatment of mCRC [3]. Importantly, it has been shown that the success of immunotherapies highly depends on the structure and composition of tumors. For example, responsiveness to immune checkpoint inhibitors (ICI) has been positively correlated with the extent of intratumoral immune cell infiltration and the overall tumor mutational burden (TMB), among other factors. In the context of CRC, immunotherapies have shown impressive efficacy in tumors with DNA mismatch repair deficiency (dMMR) and high microsatellite instability (MSI-H), but not in tumors with proficient DNA mismatch repair (pMMR) with low MSI (MSI-L), or with stable microsatellite (MSS), which represent a large majority [3,5]. Therefore, understanding the underlying molecular mechanisms of tumor immunomodulation is essential to further improve the efficacy of immunotherapies in mCRC, particularly in non-responsive patients.

**Figure 1:**
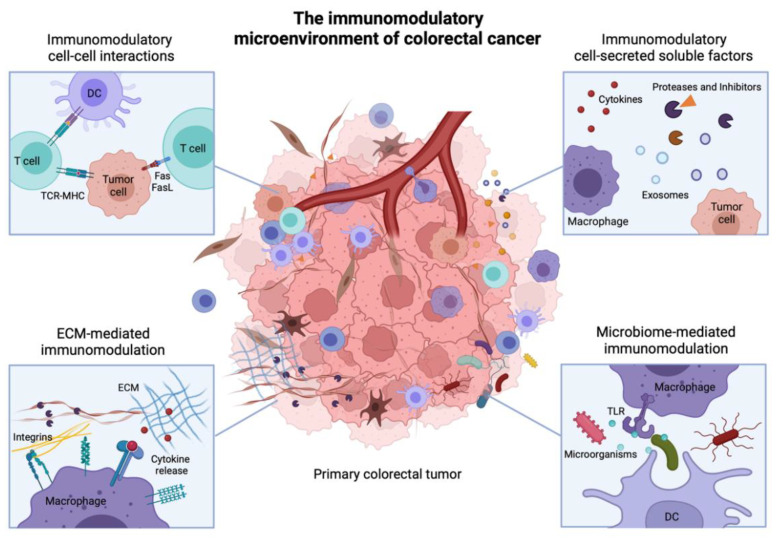
Overview of the key types of molecular interactions with immunomodulatory functions in the TME of colorectal tumors. Immunomodulation in the TME is dynamically regulated by cell-cell interactions, cell-secreted soluble factors, ECM-mediated interactions, and interactions with the microbiome (ECM: extracellular matrix; DC: dendritic cell).

In this review, we provide an overview of the key molecular mechanisms that shape anti-tumor immune responses in CRC. We first recall the main stages of disease progression and discuss the heterogeneity of immune microenvironments in colorectal tumors. We then present multiple key molecular interactions occurring in the colorectal tumor microenvironment (TME) that strongly modulate tumor inflammation. In particular, we categorize these interactions as cell-cell interactions, cell-soluble factor interactions, cell-matrix interactions and interactions with the tumor-associated microbiome (Figure 1). We finally conclude with an integrated view of the immunomodulatory TME and some perspectives on CRC therapies.

## 2. Overview of Colorectal Tumor Subtypes and Associated Immune Microenvironments

### 2.1. Main Stages of CRC Progression

**Figure 2:**
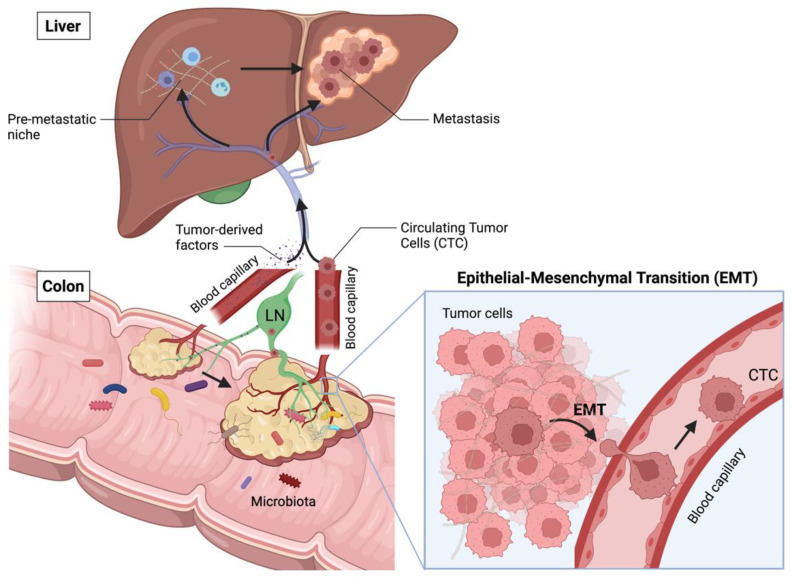
CRC progression from the primary tumor to metastasis. Primary tumor growth in the colon or rectum increases gut permeability, resulting in microbial infiltration in the tumor. In parallel, tumor-secreted factors induce the formation of PMNs in distant sites. Some cancer cells undergo EMT to intravasate and circulate as CTCs in blood or lymphatic vessels, until extravasation in a distant tissue, preferentially at the PMNs, where they form tumor metastasis upon growth (LN: tumor-draining lymph node).

CRC primary tumors generally develop in the colon or rectum, upon transformation of intestinal epithelial cells (IECs) to a benign adenoma, before progressing to malignancy upon stepwise accumulation of genetic and epigenetic aberrations (Figure 2). Cancer cells actively remodel their microenvironment by interacting with immune cells, stromal cells and the extracellular matrix (ECM). In addition, the primary tumor growth alters the integrity of the gut barrier, leading to intratumoral infiltration of bacteria and other microbes from the gut microbiome [6]. Together, immune cells, stromal cells, the ECM and the tumor microbiome constitute the TME. Additionally, the tumor releases soluble factors (e.g., cytokines, miRNA, extracellular vesicles (EVs)), uptaken at distant sites and inducing the formation of pre-metastatic niches (PMNs) [7]. PMNs form the basis for cancer metastases: they are inflamed sites in distant organs, e.g., liver and peritoneum, in which innate immune cells accumulate, such as neutrophils, monocyte-derived macrophages and dendritic cells (DCs) [8,9,10,11,12]. In addition, PMNs are characterized by increased vascular permeability, angiogenesis, matrix remodelling and immunosuppression [13], together creating a favorable microenvironment for the future implantation of metastatic cancer cells.

To disseminate, cancer cells must undergo phenotypic changes that allow them to exit their cell cluster organization, acquire the capacity to penetrate blood or lymphatic vessels, and survive into circulation. This process is referred as epithelial-to-mesenchymal transition (EMT), reflecting the loss of epithelial cell characteristics and the acquisition of a mesenchymal cell phenotype with enhanced invasive behavior. These changes importantly permit some cancer cells to intravasate, after which they become circulating tumor cells (CTCs).

CTCs can survive in circulation and attach to endothelia in distant tissues, preferentially at the PMNs. They can then extravasate, invade the parenchyma and proliferate to generate metastasis [14,15]. Because metastases frequently resemble the primary tumor genetically and phenotypically, the changes allowing a CTC to disseminate are thought to be dynamic, transient, and reversible. Therefore, cancer cells in the metastasis can re-acquire an epithelial phenotype, highlighting the high plasticity and reprogramming capacity of these cells [16].

Eventually, metastases grow and remodel their microenvironment, potentially leading to organ dysfunction and further cancer spread. In CRC, metastases generally form in primary tumor-draining lymph nodes, liver, lung, and peritoneum [17]. Intriguingly, the TME of CRC metastases have also been shown to contain a microbiome [18].

### 2.2. CRC Consensus Molecular Subgroups (CMS) and Associated Immune Landscapes

Colorectal tumors are highly heterogeneous with respect to one another. Both the tumor characteristics and the patient conditions affect the composition of the TME, subsequently dictating disease progression, responsiveness to cancer treatments and survival prognosis. Indeed, the primary tumor’s specific genetic and epigenetic alterations, its localization (i.e., ascending, transverse or descending colon, or rectum) and the hosting organs in case of metastasis strongly impact the composition of the TME [19,20,21]. Furthermore, the patient’s genetic pre-disposition, co-morbidities, bowel pre-conditions (e.g., inflammatory bowel diseases) or lifestyle (e.g., diet, physical activities, smoking) similarly alter the TME [22].

In 2015, a classification of colorectal tumors has been established to draw molecular similarities between them, to better understand tumor biology and to improve diagnosis and therapeutic strategies [22]. In total, four CMS, gathering almost 90% of all CRC tumors, have been defined. Each of these subtypes is characterized by a particular TME and a different level, type and quality of anti-tumor immune response [23].

CMS1 is hypermutated, has MSI and activates strong anti-tumor immune responses. CMS1 represents about 13% of all CRC, yet is the most deadly subtype upon relapse. Most CRC tumors with *BRAF* gene mutations belong to this subtype. CMS1 tumors are heavily infiltrated with activated CD8^+^ and CD4^+^ T cells and characterized by high expressions of major histocompatibility complexes (MHC; in humans referred as human leukocyte antigens (HLA)) and ICI molecules.

CMS2 is the canonical subtype, displaying epithelial differentiation and characterized by the activation of WNT and MYC signaling pathways. It is the most frequent CRC subtype, representing 37% of CRC. CMS2 tumors have low infiltration of immune cells, with the ones present being mostly naïve cells. In addition, they poorly express MHC and ICI molecules.

CMS3 also displays epithelial differentiation, yet it is characterized by a strong metabolic dysregulation, with increased sugar, nucleotides, and fatty acid metabolism. As such, it is referred to as the metabolic CRC subtype and represents 13% of CRC. Interestingly, *KRAS* gene mutations, which are known to alter cell metabolism, are enriched in CMS3. Similar to CMS2, CMS3 tumors have low infiltration of mostly naïve immune cells. However, they express MHC and ICI molecules.

CMS4 is known as the mesenchymal CRC subtype, due to an increased expression of genes involved in EMT, stromal infiltration, angiogenesis, and matrix remodeling. In addition, this subtype is characterized by a strong activation of TGF-β signaling. CMS4 represents about 23% of CRC and is the subtype associated with the worse overall survival prognosis. Moreover, CMS4 tumors are unfavorably inflamed, as they are infiltrated with immunosuppressive M2-polarized macrophages, regulatory T cells (Tregs) and myeloid-derived suppressive cells (MDSCs).

Importantly, responsiveness to cancer immunotherapies, particularly to ICI, has been strongly correlated with the characteristic of the CRC immune microenvironment. For example, high infiltration of activated CD8^+^ and CD4^+^ T cells, such as in CMS1 tumors, is associated with good prognosis and less tumor recurrence, although multiple mechanisms of tumor immune evasion could still make these tumors resistant to therapies [23]. In contrast, high levels of Tregs or MDSCs, such as in CMS4, rather indicate a poor prognosis. Many other immune cell types, such as DCs, natural killer (NK) cells or macrophages, can have positive or negative effects depending on their phenotypes, which can be pro- or anti-inflammatory.

Noteworthily, the immune microenvironment of CRC tumors can be modulated by cancer treatments. For example, cancer chemotherapy has been shown to induce cell necrosis that releases immunostimulatory danger signals into the TME [24] and to increase tumor infiltration by cytotoxic CD4^+^ T cells [25]. Whether such chemotherapy-induced tumor inflammation could enhance responsiveness to subsequent ICI in mCRC is currently under investigation (e.g., METIMMOX clinical trial: NCT03388190). Therefore, better understanding how to modulate the immune environment of CRC is essential to improve the effectiveness of current immunotherapies and to develop novel, more effective and safe future therapies.

## 3. Immunomodulatory Cell-Cell Interactions in the TME

In the TME, many different types of interactions control anti-tumor immune responses. Undoubtedly, cell-cell interactions account for one of the most important types. Cell-cell interactions rely on direct or indirect cell-cell contact, mediated via cell surface receptors and ligands. They can induce signaling in one or the two engaged cells (uni- vs. bi-directional signaling) to instruct behavior [26]. In addition, these interactions can occur between different cell types, as immune cells directly interact with other immune cells, with tumor cells and with stromal cells. For example, tumor cells contain multiple surface ligands and receptors that can bind to immune cells receptors, often dampening immune mechanisms and promoting tumor cell survival [27]. Here, we detail several selected examples that illustrate tumor immunomodulatory mechanisms based on cell-cell interactions.

### 3.1. Direct Cell-Cell Receptor Interactions

#### 3.1.1. MHC-T Cell Receptor (TCR) Interaction and Co-Receptors

**Figure 3:**
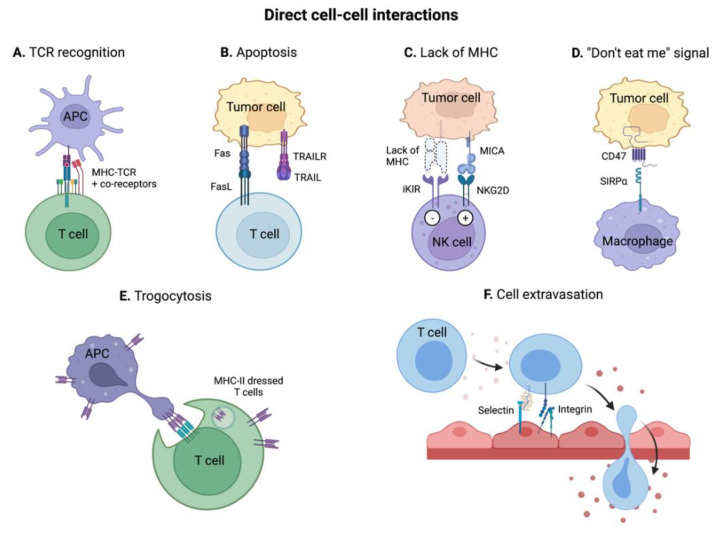
Direct cell-cell receptor interactions controlling essential immune mechanisms in the TME. (**A**) Tumor antigen recognition and education of T cells via the MHC-TCR interaction. (**B**) Apoptosis induction by cell death ligands. (**C**) NK cell activation by the lack of MHC-I or of oncogenic stress. (**D**) Phagocytosis inhibition by the expression of “do not eat me” signals by tumor cells. (**E**) Transfer of receptors from one cell to another via trogocytosis. (**F**) Extravasation of immune cell into the TME.

Tumor cells bear genetic mutations, potentially encoding for neoantigens that can be recognized by the immune system to develop antigen-specific anti-cancer immune reactions. Particularly, hypermutated CMS1 colorectal tumors bear high amounts of neoantigens [22]. Similarly, cancer cells can re-express or over-express antigens that differentiate them from healthy cells (e.g., CEA, MUC1, MAGE) [28,29], referred as tumor-associated antigens (TAAs). Both neoantigens and TAAs can be uptaken, fragmented, and presented on the class II MHC (MHC II) of antigen-presenting cells (APCs), such as DCs or macrophages. Occasionally, they can also be presented on MHC I on DCs via antigen cross-presentation. In the tumor-draining lymph nodes, APCs’ antigen-loaded MHC (pMHC) I and II engage interactions with the TCR of CD8^+^ or CD4^+^ T cells, respectively (Figure 3A). If the pMHC-TCR interaction has a sufficiently high affinity, it maturates into an immunological synapse, in which other cell-surface receptors are recruited to provide immunostimulatory or immunosuppressive co-signaling.

Well-known co-receptors involved in the activation of T cells are members of the tumor necrosis factor receptor subfamily (TNFRSF), such as the receptor pairs CD70/CD27, OX40L/OX40, 4-1BBL/4-1BB, CD40/CD40L, HVEM/LIGHT, and of the immunoglobulin superfamily (IgSF), including B7-CD28, TIM and CD2-SLAM families [30,31]. For example, the interactions between CD80 or CD86 on the APC with CD28 on T cells positively activate the T cells, as does the interaction between ICOSL and ICOS. In contrast, interactions between CD80/86 and CTLA-4 on T cells induce T cell anergy, similar to the ones of PD-L1 or PD-L2 with PD-1.

These cell-cell receptor interactions are particularly complex. Indeed, one ligand can bind to multiple receptors, with potential competing activating and inhibitory effects (e.g., immunostimulatory CD86-CD28 vs. immunosuppressive CD86-CTLA-4). In addition, receptor expression is spatio-temporally regulated; for example, T cell activation induces downregulation of CD28 and upregulation of CTLA-4. Indeed, some dMMR-MSI-H colorectal tumors that initially displayed active cytotoxic CD8^+^ T cell (CTL) responses have been shown to raise immunosuppressive molecules such as PD-1, PD-L1 or CTLA-4 to counterbalance the cytotoxic environment, inducing tumor immune evasion [32]. Additionally, the interactions between APCs and T cells can result in a bi-directional signaling, in which a reverse signaling occurs in the APC, in addition to the forward signaling in the T cell. Bi-directional signaling has been well reported for receptors from the TNFRSF family [26], yet also happens in the IgSF family. For instance, the interaction between CD80/86 and CTLA-4 on human DCs and CD4^+^ T cells has been shown to trigger the expression of indoleamine 2,3-dioxygenase (IDO) in DCs, an enzyme that suppresses T cell proliferation and induces tumor immunosuppression [33]. Lastly, such interactions not only occur between APCs and T cells; various cell types including Treg [34], tumor cells or stromal cells [35] are also capable of expressing some of the mentioned co-receptors. Antigen-presentation by “non-professional” tumor or stromal cells often result in tumor immunosuppression, as these cells generally lack the proper set of co-receptors to activate T cells but rather express co-inhibitory molecules [36].

Upon activation, T cells can further find their cognate specific antigens mounted on MHC I or II at the tumor site via TCR binding and exert various immunomodulatory effects. For example, CTLs binding to their specific pMHC I on tumor cells trigger the release of cytotoxic granules to kill the tumor cell. On the other hand, CD4^+^ T cells secrete multiple cytokines to enhance or dampen immune responses.

#### 3.1.2. Induction of Apoptosis via Fas/FasL and TRAIL/TRAIL-R Interactions

Another important mechanism mediated by direct cell-cell interaction in the TME is the killing of tumor cell by T cells via induction of apoptosis (Figure 3B). Tumor cell apoptosis can be triggered by the respective binding of FasL or TRAIL present on T cell surface, to the death receptors Fas or TRAIL-R on the tumor cell [37]. While this potentially limits tumor growth, it has been found that colorectal cancer cells can become resistant to T-cell induced apoptosis [38]. Even more, studies have shown that CRC cells can upregulate FasL during tumorigenesis, which in turn can trigger apoptosis of T cells, thereby repressing anti-tumor T cell responses [38,39].

#### 3.1.3. Detection of the Lack of MHC I and of Oncogenic Stress by NK Cells

One way tumor cells escape from CTL-mediated recognition and killing is by downregulating MHC I. Nevertheless, an absence of MHC I on tumor cell surface can also be detected by NK cells, similarly resulting in the release of cytotoxic granules and subsequent tumor cell death (Figure 3C). This cytotoxic mechanism also relies on cell-cell interaction between NK cells and tumor cell receptors. NK cells express both inhibitory receptors (e.g., iKIR) and activating receptors (e.g., NKG2D, NKp46). MHC I is the main ligand for iKIR and inhibits NK cells activation upon binding; thus, the lack of MHC I activates NK cells. In parallel, the activating receptors activate NK cells upon binding to stress-induced ligands, present at the surface of tumor cells (e.g., MICA/B, RAET-1G). These ligands are not generally expressed in healthy cells but are upregulated during carcinogenesis [40,41]. Ultimately, the release of cytotoxic granules depends on the dynamic integration of activating and inhibiting signals.

#### 3.1.4. “Do Not Eat Me” Signals: Escaping Phagocytosis

Phagocytosis is a central innate immune mechanism by which macrophages and other phagocytic cells clear dying cells from tissues. Due the cellular and microenvironmental dysregulation, many tumor cells enter apoptosis and express pro-phagocytic (“eat me”) signals, to instruct the macrophages to destroy them. Particularly, calreticulin, one of the major pro-phagocytic signals, is upregulated in multiple human cancers including in MSI-H CRC [42,43]. On the other hand, tumor cells can also protect themselves from phagocytosis by expressing the cell surface receptor CD47, known as an anti-phagocytic (“do not eat me”) signal (Figure 3D). CD47 efficiently suppresses phagocytosis when bound to the signal regulatory protein α (SIRPα) on macrophages. It is the balance between pro- and anti-phagocytic signals that determines the initiation of phagocytosis. High expression of CD47 in colorectal tumor cells has been shown to prevent them from being cleared by innate immune cells [44,45]. In addition to CD47, the MHC I component β2-microglobulin and CD24 have also been shown to act as potent “do not eat me” signals, upon binding to their respective inhibitory receptors LILRB1 and Siglec-10 on tumor-associated macrophages (TAMs) [46,47].

Interestingly, immune checkpoint blockade of anti-phagocytic cell-cell interactions is emerging as a promising anti-cancer immunotherapeutic approach, complementary to T-cell activating immunotherapies [48].

#### 3.1.5. Trogocytosis: Receptor Transfer between Tumor and Immune Cells

Trogocytosis is a mechanism by which a cell endocytoses a portion of another cell membrane and re-exposes it at its surface, therefore acquiring receptors from the donor cell (Figure 3E). Trogocytosis is also mediated by strong physical cell-cell interactions, allowing membrane uptake from the donor cell. Using this process, colorectal tumor cells have been shown to acquire immune receptors (e.g., CD45 and CD4) and functional immunoregulatory molecules (e.g., Tim3, CTLA-4 and PD-1) from T cells, thus enhancing tumor immunosuppression [49]. Surprisingly, the immunostimulatory receptors CD80 and CD86 have also been detected on colorectal tumor cells, potentially derived from previous trogocytoses during which T cells acquired them from APCs [49,50]. In fact, trogocytosis was initially discovered due to membrane transfers between T cells and APCs, resulting in T cell acquisition of MHC II molecules [51].

#### 3.1.6. Immune Cell Recruitment in the TME

Another mechanism by which direct cell-cell interactions affect inflammation is by regulating immune cell infiltration into the tumor or in PMNs (Figure 3F). Indeed, initial local inflammation increases vascular permeability and activates endothelial cells, which upregulate specific ligands to interact with the circulating leukocytes. The transmigration of leukocytes across the vascular wall then follows several well-defined steps including the rolling, adhesion, and transmigration (i.e., diapedesis). Importantly, the type of leukocytes that are recruited depends on whether their cell surface ligands have cognate receptors on the endothelial cells [52]. For example, PSGL-1 and CD44 on neutrophils or activated T cells bind to E-selectin on the endothelial cells, which favors their rolling on the endothelium and slows their velocity. Next, the leukocytes tightly bind to adhesion molecules such as ICAM and VCAM on endothelial cells, allowing their arrest at the inflamed site. Notably, the integrins LFA-1 or Mac present on lymphocytes, neutrophils and monocytes strongly adhere to ICAM-1 [53]. Lastly, leukocytes transmigrate between or across endothelial cells by binding to integrins or other adhesion molecules (e.g., PECAM-1). Of note, not only immune cells extravasate but also cancer cells during the formation of metastasis [54].

### 3.2. Indirect Cell-Cell Receptor Interactions

Indirect cell-cell interactions occur when two cell-surface receptors are connected via a third (or more) molecule. Multiple innate immune mechanisms are based on such tri-partite interaction, in which the third bridging molecule displays dual affinity for each of the receptors to connect. For example, antibody-mediated cell-cell interactions or immune-mediated clearance of apoptotic debris/cells involve indirect cell-cell interactions.

#### 3.2.1. Antibody-Mediated Cell-Cell Interactions

**Figure 4:**
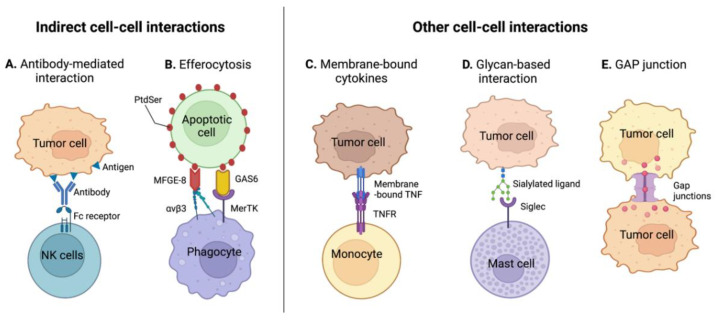
Indirect cell-cell receptor interactions and other types of molecular interactions modulating immune responses in the TME. (**A**) Antibody-dependent interaction between tumor antigens and Fc receptors of immune cells. (**B**) Recognition of PtdSer by phagocytes via the bridge proteins MFGE-8 and GAS6. (**C**) Direct cell-cell interaction via membrane-bound cytokines. (**D**) Cell-cell interaction via glycan recognition. (**E**) Cell-cell interaction via proteins involved in cellular communication (e.g., gap junction).

Antibody-dependent cytotoxic mechanisms are immune processes capable of killing cancer cells. Among them, antibody-dependent cell cytotoxicity (ADCC) and antibody-dependent cell phagocytosis (ADCP) involve indirect cell-cell interactions (Figure 4A) [27]. Upon tumorigenesis, B cell immunity can raise and lead to the production of immunoglobulin G (IgG) antibodies, specific to tumor cell surface-exposed neoantigens. These antibodies can on one side bind to their specific neoantigen on tumor cell surface, via their variable regions, while on the other side bonding to immune cells via their constant region, therefore creating a molecular bridge between the tumor and the immune cell. In the case of ADCC, the tumor cell-bound antibody interacts with Fc receptor γ (FcγR) present on NK cells, particularly FcγRIIIa, which induces the release of cytotoxic granules inducing cancer cell death [55]. In contrast, interactions with the FcγR IIa and IIIa on macrophages initiate phagocytosis of the tumor cells, referred as ADCP [56].

While ADCC and ADCP naturally occur in some colorectal tumors, a strong interest has raised to understand whether clinical monoclonal antibodies (mAb) are capable of inducing ADCC against tumors. For example, several studies have shown that the anti-epidermal growth factor receptor (anti-EGFR; Cetuximab), used for the treatment of RAS wild-type mCRC, can mediate ADCC in vitro in an EGFR-dependent concentration, and more impressively, independently of the RAS status of the tumor [57,58]. Nevertheless, the limited in vivo efficacy on RAS-mutated mCRC has fostered the engineering of ADCC-promoting anti-EGFR. Gerdes et al., for example, developed a glycol-engineered anti-EGFR named GA201 that outperforms Cetuximab efficacy in wild-type and RAS-mutant mCRC in pre-clinical xenograft mouse models [59].

#### 3.2.2. Tumor Efferocytosis

The recognition and clearance of apoptotic cells and debris by phagocytes, referred as efferocytosis, similarly involve indirect cell-cell interactions (Figure 4B). On the outer leaflet of cell membranes, the presence of phosphatidylserine (PtdSer) phospholipids is a signal for phagocytosis initiation. PtdSer is a ligand for multiple macrophage receptors, some of which needing a bridge protein to attach to PtdSer. For example, the milk fat globule-EGF factor 8 (MFGE-8) possesses a domain that binds to PtdSer and another one that interacts with the integrins α_V_β_3_ or α_V_β_5_ expressed on macrophages. Similarly, the growth arrest-specific 6 protein (GAS6) or the protein S (PROS1) are necessary to bridge PtdSer to receptor tyrosine kinases (RTKs) on macrophages to trigger phagocytosis [60,61]. Upon tumor efferocytosis, macrophages secrete immunosuppressive cytokines to create a tolerogenic environment, and polarize toward an M2-phenotype. DCs, which also have phagocytic activities, similarly acquire a more immunosuppressive phenotype, ultimately impairing T cell anti-tumor immune responses [62]. While the role of MFGE-8 and GAS6 is under investigation in CRC [63,64], regulating efferocytosis in cancer appears as an emergent therapeutic strategy [65].

### 3.3. Other Immunomodulatory Cell-Cell Interactions

Lastly, many other immunomodulatory cell-cell interactions occur in the TME, some differing from the classical receptor-receptor contact. Indeed, certain molecules that are generally secreted in the TME have isoforms that are produced as membrane-anchored proteins, capable of directly interacting with their cognate receptor on another cell membrane (Figure 4C). For example, the membrane-anchored tumor necrosis factor-α (mTNFα) has a high affinity to the TNF receptor 2 (TNF-R2) and has been shown to alter the survival of monocytes in tumors, potentially leading to their depletion [66,67].

Additionally, cell-cell interactions not only consist of protein–protein interactions, but can also take place between lipids and proteins, as illustrated by PtdSer during efferocytosis, or between glycans and proteins. For example, Siglec receptors (sialic acid-binding immunoglobulin-type lectins) are mostly found on immune cells and strongly interact with glycoproteins and glycolipids containing the sialic acid glycans (Figure 4D) [68]. For instance, Siglec-6 has been shown to reduce degranulation of mast cells and alter cytokine secretion upon interaction with colorectal cancer cells [69].

Finally, the diversity of pathways that can influence immune responses in the TME have foster the exploration of immunomodulatory roles of other protein families, for example, of ephrins or gap junction proteins, which are widely involved in cell-cell communication (Figure 4E) [70,71].

In conclusion, cell-cell contacts via surface molecules regulate central immune mechanisms in the TME. These contacts are necessary to mount anti-tumor immune responses yet are also involved in immunosuppression and tumor escape, depending on the nature of the receptors at play and the cell types in contact. From a clinical perspective, blocking of cell-cell interactions has so far been one of the most exploited strategies for the development of immunotherapies. For example, anti-PD-1 (e.g., Nivolumab) and anti-CTLA-4 (e.g., Ipilimumab) are currently first-line treatments in multiple cancers and are approved for use in dMMR and MSI-H in mCRC [72]. On the other hand, engagement of cell-cell interactions is also being extensively addressed, as through the development of bispecific T cell engagers (BiTE), for instance, which provides antibody-like bridge proteins connecting T cells to tumor cells to elicit tumor cytotoxicity.

## 4. Immunomodulatory Soluble Factors in the TME

While cell-cell interactions are key regulators of immune responses in the tumor, cells secrete many soluble factors that complementarily orchestrate immune responses. In some cases, these soluble signals—particularly cytokines—are required for the maturation of immune responses. In fact, all cell-cell interactions presented above take place in presence of cytokines. Soluble immunomodulatory factors can be released in the TME by controlled secretion from immune, tumor or stromal cells, or by uncontrolled release during cell death. Moreover, some factors are secreted in an inactive form and necessitate further processing to become active. In this section, we summarize important categories of immunomodulatory soluble signals present in the TME, including cytokines, proteases, soluble receptors, nucleic acids, amino-acids, and reactive oxygen/nitrogen species.

### 4.1. Cytokines

**Figure 5:**
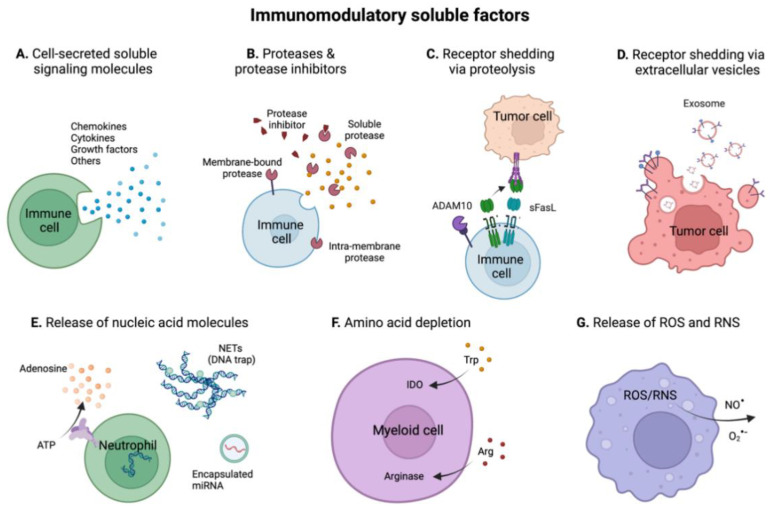
Cell-secreting soluble factors as potent immunomodulatory molecules in the TME. (**A**) Cell secretion of potent immunomodulatory signaling proteins (e.g., chemokines and cytokines). (**B**) Immune regulation via the secretion of protease and protease inhibitors. (**C**) Proteolytic release of cell surface-bound immunomodulatory receptors or factors. (**D**) Receptor shedding in the TME by exosomes or other EVs. (**E**) Immunomodulation via the presence of nucleic acid in the TME (e.g., ATP, adenosine, miRNA, NETs). (**F**) Depletion of amino acids from the TME by amino-acid degrading enzymes (e.g., IDO, arginase). Amino acids are essential to some immune cells’ functions. (**G**) Release of ROS and RNS upon cellular stress. ROS and RNS react and damage DNA, proteins, lipids and glycans, thereby positively or negatively affecting immune responses.

Cytokines are the most potent cell-secreted soluble proteins that regulate immune responses. They are mainly produced by immune cells, although tumor or stromal cells can secrete them, and operate via autocrine, paracrine, or endocrine signaling by direct binding to cell surface receptors (Figure 5A). Cytokines can have broad immunostimulatory or immunosuppressive effects, or have mixed functions depending on the targeted cell type. While having primary functions on immune cells, they additionally affect tumor and stromal cells, resulting in overall pro- or anti-tumorigenic effects on the primary tumor or on metastasis development.

Well-known families of cytokines include chemokines, interleukins (ILs), interferons (IFNs), the TNF superfamily, colony-stimulating factors (CSFs) and the transforming growth factor-β (TGF-β) superfamily. Together, more than 100 cytokines and isoforms have been discovered [73]. Importantly, many cytokines have been assessed or are under investigation in clinical trials as immunotherapeutics for colorectal cancer (Table 1).

#### 4.1.1. Chemokines

Chemokines are involved in the regulation of immune cell chemotaxis and trafficking. They importantly instruct the recruitment of monocyte/macrophages, DCs and lymphocytes from the blood vasculature into the tumor and their migration within the tumor. In addition, chemokines control the migration of APCs from the tumor to lymphoid tissues [74,75]. These migratory immune cells can be pro-inflammatory, in case of activated CTLs, or immunosuppressive, such as MDSCs or Tregs. Chemokines are commonly divided into four main families, the CXCL, CCL, CX_3_CL and XCL families. In total, approximately 50 chemokines and 20 chemokine receptors have been identified in humans, with some chemokines capable of binding to multiple receptors, and vice-versa, in a redundant way [74,76].

Chemokines usually function by forming a concentration gradient from the source cell which secretes it to the recipient cells, on which they bind to their cognate G-protein coupled receptors (GPCR). GPCR are uniformly distributed on the recipient cell surface, which allows the detection of chemotactic gradients from any direction. Upon chemokine binding, activation of GPCR permits the recipient cell to sense differences in chemokine concentration across its diameter, leading to cell polarization. The recipient cell then migrates toward or away from higher chemokine concentrations, in the respective cases of chemoattractive or chemorepellent chemokines [77,78]. For example, effector T cells upregulate the expression of CXCR3 upon activation, making them capable of detecting CXCL9/10/11 gradients produced by immune and stromal cells upon inflammation, which result in T cells recruitment into the TME [79,80]. As another example, DC trafficking from the tumor to the draining lymph node is driven by the secretion of CCL21 by lymphatic endothelial cells [81], which creates a gradient in the perilymphatic interstitium. CCL21 gradients attract DCs from the tumor intertitium to the lymphatic vasculature upon binding to the receptor CCR7. Likely using a similar mechanism, it has been found that some colorectal tumor cells can express CCR7, which expression correlates to the presence of metastasis in the regional lymph nodes [82].

In addition to spatial chemokine gradient detection, it has been recently shown that some cells respond to temporal gradients. Indeed, Aronin et al. demonstrated that myeloid cells, notably DCs and neutrophils, need to sense an increasing absolute concentration of the chemokine CCL19 and CXCL12 in order to have persistent directional migration toward it, which particularly occurs during initial gradient formation. In contrast, established stable gradients failed to induce persistent migration of these cells.

Finally, it is worth noting that some atypical GPCRs function as decoy or scavenger receptors for chemokines, instead of inducing cell signaling and migration [83]. These receptors still modulate immune responses by altering the bioavailability of chemokines.

#### 4.1.2. ILs

ILs are a subgroup of cytokines that work as soluble immune messengers to primarily modulate survival, growth, differentiation, and activation of immune cells during inflammation. Nevertheless, they additionally exhibit diverse effects on a variety of cell types in the tumor [84]. There are currently 41 ILs, most of them classified in about eight families based on their genomic organization, structural homology or receptor-binding properties. Their individual roles in cancer, and more specifically in CRC, were very well reviewed in [85,86,87]. ILs generally act by paracrine or autocrine signaling on cells in the local microenvironment.

ILs bind to their cognate receptors exposed on the cell surface with various affinities depending on the subunits composing the cytokine or the receptors. For example, IL-2 is a monomer which binds to multiple receptors, with low affinity to the IL-2Rα monomeric receptor, moderate affinity to the IL-2Rβ/γC heterodimeric receptor and with high-affinity to IL-2Rα/β/γ trimeric complex, with only the latter two being capable of inducing cell signaling [87,88]. Interestingly, T cells upregulate the expression of IL-2Rα during activation to be more responsive to low concentration of IL-2, which promotes their proliferation. In contrast, Treg naturally express the three subunits, thus being a high-affinity receptor for IL-2 [87,88]. IL-2 was the first approved interleukin for cancer immunotherapy, and many clinical trials explore its efficacy in combination therapy for colorectal cancer (Table 1).

Similarly, IL-12 displays different affinities and biological effects upon binding to its receptor IL-12R, although in this case, it is the composition of IL-12 that dictates its effects. Indeed, the IL-12 heterodimer called IL-12p70 is highly active and induces the secretion of the pro-inflammatory cytokine IFNγ upon signaling in T and NK cells. In contrast, the monomer or homodimer IL-12p40 competitively binds to IL-12R, but without triggering IFNγ production [89]. Importantly, IL-12p70 is the most potent cytokine for inducing naïve CD4^+^ T cell differentiation toward T helper 1 (Th1) cells, promoting a strong cytotoxic adaptive immunity, and to polarize macrophages toward a pro-inflammatory M1 phenotype, both of which correlating with good prognosis in CRC [90]. IL-12 has also been the focus of multiple clinical trials in CRC (Table 1), but has been generally associated with high toxicity [91].

Furthermore, the high modularity in the molecular composition of ILs and IL receptors allows many IL receptors to interact with multiple ILs. For instance, the type II IL-4 receptor (IL-4R) binds to both IL-4 and IL-13. IL-4 signaling importantly promotes the polarization of macrophages toward an immunosuppressive M2-like phenotype [92]. In addition, IL-4 signaling drives the differentiation of naïve CD4^+^ T cells into T helper 2 (Th2) cells [93]. This Th2-biased immune response is generally considered as being not optimal to fight tumor cells as compared to the cytotoxic Th1 response [90,94]. Nevertheless, the survival prognosis associated with the Th2 response in CRC is not as poor as the one associated with Th17 responses. The Th17 phenotype is characterized by the secretion of high levels of IL-17 upon T cell activation [90,95]. These examples highlight one key role of ILs in instructing the different types of immune responses.

#### 4.1.3. IFNs

IFNs were originally defined based on their roles in interfering with viral replication, yet they have many important functions in cancer. There are three types of IFNs, among which type I and II are the most characterized.

The type I and III IFNs are secreted upon cell detection of danger- or pathogen-associated molecular patterns (DAMPs and PAMPs respectively). In tumors, cell stress and death induce the release of DAMPs, such as the high mobility group box 1 (HMGB1) protein, heat shock proteins, calreticulin or high extracellular ATP [96,97]. In addition, the microbiome of colorectal tumors also presents PAMPs. Stimulation of pattern recognizing receptors (PRRs) by DAMPs and PAMPs induces the production of type I IFNs by the stressed or dying cell and by its neighbors. Notably, IFNβ can be expressed by most cell types upon PRR activation [98]. Type I IFNs include 16 members, 12 IFNα, IFNβ, IFNε, IFNκ and IFNω, all being monomeric cytokines binding to the heterodimeric receptor IFNAR [99]. They have been shown to modulate innate and adaptive immune responses, particularly by increasing pro-inflammatory cytokine secretion and antigen presentation by APCs, by enhancing NK and T cells cytotoxic functions and immunological memory, and by dampening Treg-mediated immunosuppression [100]. Importantly, they have direct effects on tumor cell proliferation, particularly by arresting cell cycle and promoting senescence or apoptosis [101]. IFNα was the first cancer immunotherapy approved by the U.S. FDA in 1986 [102], although not for CRC, yet has been studied in many CRC-inclusive clinical trials (Table 1). On the other hand, type III IFNs are similar to type I IFNs but mainly act in mucosal tissues, being potentially highly relevant in CRC. They have been discovered more recently and currently comprise 4 IFNλ members.

In contrast, the only type II IFN, IFNγ, is secreted by immune cells, notably T and NK cells, in response to stimulation by other cytokines (e.g., IL-12, type I IFNs) [103]. IFNγ is a homodimer and interacts with the heterodimeric receptor IFNGR. It is the main effector cytokine that induces Th1 immune responses while inhibiting Th2 and Th17 responses. IFNγ has been shown to increase M1 macrophages, MHC expression and antigen presentation on APCs, T and NK cell cytotoxicity. It also has anti-proliferative effects on tumor cells, similar to type I IFNs [103,104]. Nevertheless, tumor cells can lose responsiveness to IFNγ by loss of IFNGR and mutations in the IFNγ signaling pathway, which participates in tumor immune evasion. IFNγ has also been included in CRC clinical trials (Table 1), and constitutes a potential target for CRC immunotherapy [105].

#### 4.1.4. TNF Superfamily (TNFSF)

TNFSF are a subgroup of about 20 homotrimeric transmembrane proteins that can be proteolytically cleaved from the cell membrane to act as cytokines, although they can also function by direct cell-cell interactions. They interact and signal to about 30 different receptors, called TNFSFR. TNFSF regulate central pro-inflammatory and anti-tumor immune mechanisms, for instance by providing co-stimulatory signals during immune activation or by triggering cell death in targeted cells [106,107,108]. Indeed, the co-stimulatory receptors CD70, OX40L, 4-1BBL, CD40L, LIGHT involved in T cell activation, as well as the FasL and TRAIL involved in cell apoptosis, are part of the TNFSF.

Additionally, the well-known TNFα is part of this family. TNFα is a major pro-inflammatory cytokine named after the observation that it induces rapid hemorrhagic necrosis in tumors at high dose, mediating tumor shrinkage [109,110]. Therefore, it has been tested as an immunotherapy in cancer, including in CRC (Table 1), but has not reached the clinic. In fact, it has also been found that endogenous levels of TNFα have some pro-tumorigenic effects by promoting infiltration and functions of immunoregulatory cells, including MDSCs and Treg, and by enhancing tumor cell survival and metastatic potential [110,111]. Serum levels of TNFα in CRC correlate with advanced stages of the disease and worst overall survival [112].

#### 4.1.5. CSF Superfamily

CSFs are factors that induce survival, proliferation or differentiation of hematopoietic progenitors and immune cells. There are four CSFs: GM-CSF signals to granulocytes and macrophages, G-CSF to granulocytes, M-CSF to macrophages and a multi-CSF (being IL-3) that affects a large range of hematopoietic cells. That said, their effects are not completely restricted to a single cell type; for example, M-CSF can stimulate granulocytes colony formation from some progenitor cells [113,114]. CSFs are expressed by a variety of cells, such as macrophages, T cells, endothelial cells, fibroblasts and some tumor cells, during inflammation. They are locally secreted and mostly act in a paracrine fashion, although they can enter the blood circulation for endocrine signaling. In particular, they are involved in the recruitment of circulating neutrophils, monocytes and T cells, the activation of DCs and macrophages, and importantly regulate the renewal of immune cell populations [114,115]. In CRC, GM-CSF has been shown to correlate with improved survival [116], although GM-CSF upregulation during colitis promotes malignant transformation into CRC [117]. GM-CSF has been widely assessed in the clinic for CRC, both in a colon cancer cell-secreting GM-CSF vaccine, known as GVAX, and as a recombinant protein (Table 1).

#### 4.1.6. TGF-β Superfamily

The TGF-β superfamily contains about 33 cytokines that are structurally related. There are five families included in the TGF-β superfamily, with diverse roles in tissue regulation, growth and differentation, during homeostasis, inflammation and cancer [118,119]. Among them, the TGF-β family contains the three isoforms TGF-β1, -β2 and -β3, with TGF-β1 being the most abundant and ubiquitous variant. TGF-β is secreted in an inactive form, further requiring activation by proteases or cell-induced release from the ECM (as discussed in Section 5.2). It is one of the most potent immunosuppressive cytokine and drives immune escape. Indeed, it can suppress lymphocytes responses, particularly by inhibiting the effector functions of CD8^+^ T cells and NK cells, preventing differentiation of Th1 and Th2 cell responses, and promoting Th17 or Th9 responses and the development of Treg. It also inhibit B cells proliferation. On innate immune cells, TGF-β promotes tolerogenic phenotypes of DCs and M2 phenotypes in macrophages. Lastly, TGF-β also modulates the cytokines secretion of immune cells [119]. Of note, CMS4 CRC is characterized by TGF-β expression. As a potential clinical therapy, TGF-β traps have been developed to reduce TGF-β-mediated immunosuppression in cancer patients and are currently tested in clinical trials (Table 1).

#### 4.1.7. Other Cell-Secreted Soluble Signaling Proteins

In addition to cytokines, many other soluble signaling proteins can display direct or indirect immunomodulatory effects in the TME, such as growth factors or hormones. For example, the vascular endothelial growth factors VEGF-A and VEGF-C can respectively promote angiogenesis and lymphangiogenesis in the TME, offering new routes for immune cell trafficking. In addition to cells, tumor lymphatics also drain tumor-derived immunosuppressive soluble signals to the tumor-draining lymph nodes, modulating the development of adaptive immune responses. Additionally, VEGF-C can directly signal to macrophages to polarize them toward immunosuppressive cells and promote colorectal tumor growth [120].

As another example, leptin is a hormone well known for its role in regulating food intake and its involvement in obesity, a risk factor for the development of CRC. In tumors, leptin can drive accumulation of MDSCs [121], while on the other hand modulating the releases of pro-inflammatory cytokines (e.g., IL-8) and promoting T cell immunity [122].

### 4.2. Proteases and Protease Inhibitors

The presence of proteases and proteases inhibitors in the TME strongly regulates immune mechanisms, notably by regulating the bioavailability of signaling proteins and by remodeling the microenvironment. Multiple families of proteases exist, being secreted in the extracellular space (e.g., metalloproteinases, serine proteases and cysteine proteases), bound to cell membranes, intercalated in the membrane lipid bilayer or present intracellularly (Figure 5B) [123].

While not generally having direct effects on immune cell signaling, they importantly control the activity of cytokines and other signaling molecules. Indeed, some proteins are expressed with propeptides that inactivate them until proteolytic processing. For example, multiple IL-1 members, which are importantly involved in the initiation and amplification of immune responses, are expressed with an N-terminal propeptide. Cleavage of this propeptide by caspase 1, neutrophil elastase or mast cell-derived chymase induces conformational changes in the cytokine and makes it bioactive [124,125]. On the other hand, extracellular proteases can regulate half-life of proteins by degrading them and limiting exposure to cells [123]. For instance, IL-6 and IL-13 can be proteolytically degraded by cathepsin G and chymase, which limits their activity [126]. Importantly, activation and degradation of cytokines by proteases can be counter-balanced by the production of endogenous protease inhibitors [126].

In addition, extracellular proteases can also mediate cytokine release from the ECM or from cell membranes (discussed in part 4.3). For example, CCL21 has a highly positively charged C-terminus domain that strongly interacts with GAGs in the ECM, importantly involved in gradient formation. This domain is highly sensitive to DC-secreted proteases and to plasmin. Cleavage and release of CCL21 from the matrix upon contact with DCs makes the chemokine bioavailable to the cells. Interestingly, such mechanism of protease-mediated chemokine release from the ECM could amplify local chemokine gradient to better guide DCs to the lymphatic vessels [127,128]. Similarly, this process can further be regulated by protease inhibitors [129,130].

Moreover, the balance between proteases and inhibitors regulates the degradation of the ECM directly, particularly by matrix metalloproteinases (MMPs) and the tissue inhibitors of metalloproteinases (TIMPs). Indeed, ECM breakdown by MMPs allows infiltration, migration, and other activities of immune cells in the tumor, among other things. For example, MMP-8, produced by neutrophils, is associated with sustained inflammation in the TME, although being pro-tumorigenic [131,132]. Likewise, dysregulation of TIMPs in cancer has strong impacts on tumor progression and inflammation [133]. Besides MMPs, other well-known families of proteases are the disintegrin and metalloproteinases (ADAMs), and the ADAMs with thrombospondin domain (ADAMTSs) [132].

Finally, there are emergent roles for proteases in inducing direct signaling on tumor cells or antimicrobial activities, independently of their proteolytic functions. Nevertheless, little is known about whether these are relevant in tumor immunomodulation [134].

### 4.3. Receptor Shedding in the TME

Some important immunomodulatory cell-surface receptors are known to have soluble isoforms or can be solubilize by protease-mediated cleavage and release of their ectodomain. This is the case of multiple receptors from the TNFSFR, such as the TRAIL or FasL receptors. Indeed, soluble form of FasL is generated upon proteolytic cleavage by ADAM10 (Figure 5C) [135,136]. Soluble FasL (sFasL) activates Fas on target cells to trigger apoptosis. For example, T cells can be depleted by the release of sFasL by colon adenocarcinoma cells, a mechanism potentially involved in tumor immune escape [137]. Interestingly, it has been further shown that the intracellular part of FasL can be additionally cleaved by SPPL2a, an intramembrane cleaving protease, generating a small fragment that can translocate into the nucleus to directly modulate gene expression.

Another way by which cells shed receptors in the TME is via the release of EVs, including exosomes (Figure 5D). For example, exosomes have been shown to transport antigenic pMHC complexes, immunoregulatory receptors or integrins [138]. In addition to shedding membrane-bound receptors, EVs contain soluble proteins, different types of RNA (e.g., mRNA, miRNA) and lipids that can be delivered to a targeted cell [139]. In CRC, tumor-derived exosomes can modulate anti-tumor immune responses by enhancing Treg and MDSCs recruitment, dampening NK and T cells activities, and promoting M2-macrophages, as reviewed in [140].

### 4.4. Immunomodulation by Nucleic Acids in the TME

The TME not only contains immunomodulatory proteins, but also nucleic acids that affect anti-tumor immunity (Figure 5E). Particularly, high concentration of extracellular ATP in tumors, of about 1000-times higher than in healthy tissues, acts as a pro-inflammatory danger signal that activates innate and adaptive immune responses [141]. Nevertheless, extracellular ATP is rapidly hydrolyzed to extracellular adenosine, which rather displays immunosuppressive properties, also shown in CRC [142,143]. Indeed, adenosine inhibits infiltration and immune functions of T cells, NK cells, DCs, macrophages and neutrophils, while promoting immunoregulatory responses of Treg and M2 macrophages, as well as expansion of MDSCs [142].

Other nucleic acids that modulate anti-cancer immune responses are RNAs, such as microRNA (miRNA). Although miRNAs are produced intracellularly, they can get encapsulated into EVs, shed in the TME and be uptaken by local or distant cells. MiRNA expression is largely dysregulated in CRC and displays immunomodulatory functions [144]. For example, miRNA-21-5p is enriched in CRC-derived EVs and induces proinflammatory responses in macrophages by binding to TLR-7, also at distant sites where it participates in the establishment of PMNs [145].

The TME also contains extracellular DNA traps released by neutrophils during process called NETosis [146]. As central players during innate immune response, neutrophils infiltrate colorectal tumors starting at early stage of inflammation, attracted by CXCL-1, -5 or -8 chemokines, which induce NETosis via binding to CXCR1 and CXCR2 [147,148]. Upon release, NETs shield tumor cells and prevent contact with cytotoxic NK and CD8+ T cells, overall protecting them from immune destruction and allowing further growth and metastasis [148,149].

### 4.5. Immunomodulation by Depletion of Amino Acids in the TME

The dysregulation of amino acids metabolism in tumors strongly affects immune responses in the TME. Particularly, abnormal expression of amino acids-degrading enzymes can deplete some amino acids that are essential for immune functions (Figure 5F) [150]. Particularly, the depletion of arginine (Arg) by arginase has been shown to inhibit T cell activation and proliferation. In tumors, arginase is upregulated in myeloid cells upon exposure to certain cytokines [151]. In parallel, the reduction of Arg concentration limits the production of nitric oxide (NO) by inducible nitric oxide synthase (iNOS), which has tumoricidal and anti-microbial activities.

Similarly, depletion of tryptophane (Trp) in the TME was observed in response to overexpression of IDO in myeloid and tumor cells. Trp catabolism induces potent immunosuppression, also inhibiting T cell effector responses while enhancing Treg [150,152].

### 4.6. Reactive Oxygen and Nitrogen Species (ROS and RNS)

In the tumor, ROS and RNS are released and accumulate due to dysregulated cell metabolism and hypoxia (Figure 5G). ROS and RNS are highly reactive molecules that react with DNA, proteins, lipids and glycans in a destructive way [153,154]. ROS have been shown to widely affect innate and adaptive immune responses in cancer, by acting as a chemoattractant for immune cells and as a regulator of phagocytosis, NETosis, macrophage polarization and lymphocyte cytotoxic responses, among others [155]. For example, Chen et al. showed that ROS produced by colorectal cancer cells induce M2 macrophages, which can be re-polarized toward M1 macrophages by ROS inhibitors [156].

In conclusion, the TME contains a tremendous amount of immunomodulatory soluble signals that act in concert with direct cell-cell interactions to build proper immune responses. In addition to the molecules discussed here, many other types of molecules affect tumor inflammation, such as bioactive lipids (e.g., prostaglandin-E2) [157] or glycans [158]. The multitude and diversity of these soluble immunomodulatory signals create a highly complex and dynamic immunomodulatory TME, with immune outcomes ranging from tumor eradication to tumor evasion.

## 5. ECM-Mediated Tumor Immunomodulation

More than cells and soluble molecules, the TME is composed of an ECM. The ECM is known to tightly regulate cell behaviors by providing cell-adhesion sites, controlling spatiotemporal release of cell-secreted factors, presenting matrix-bound factors and providing bioactive ligands [159]. The structure and composition of the ECM thus affects cell survival, adhesion, migration, proliferation and differentiation. While most studies have focused on the role of the ECM during tumorigenesis, importantly highlighting its effects on tumor growth, invasion and metastasis, a recent interest has emerged to understand its effects on anti-tumor immune responses. Here, we summarize key immunomodulatory functions of the ECM with selected examples in colorectal tumors.

### 5.1. The ECM as an Immunomodulatory Biomechanical Scaffold

**Figure 6:**
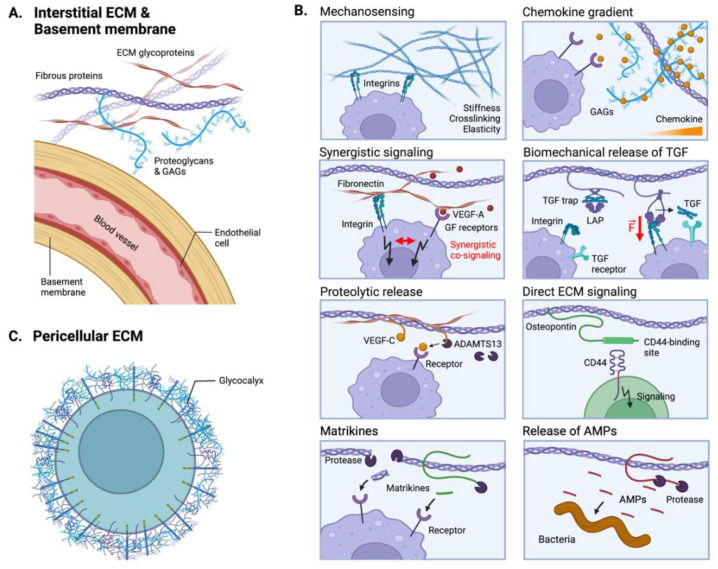
The immunomodulatory role of the ECM and examples of associated molecular mechanisms. (**A**) Schematic view of the interstitial ECM and the basement membrane localization in tissues and key components. (**B**) Example of immunomodulatory mechanisms involving interactions with the ECM. The ECM has key roles in transducing mechanical signals into cells, creating chemokine gradients and modulating bioavailability and signaling of bioactive proteins. The ECM additionally releases bioactive domains upon cleavage, directly signaling immune cells to modulate their behavior or acting as anti-microbial peptides (AMPs). (**C**) The pericellular ECM called the glycocalyx, present on every cell, is involved in the regulation of immune functions and extravasation of cells into the tumor.

The primary role of the ECM is to provide a biomechanical scaffold for cells. In the tumor, the ECM is mainly produced by fibroblasts, although tumor cells, endothelial cells and immune cells can also participate in ECM deposition [160]. In addition to secreting the ECM, cells constantly remodel the surrounding matrix via the secretion of proteases and protease inhibitors (e.g., MMPs, TIMPs). Consequently, the ECM provides a heterogeneous and highly dynamic environment, with variations in composition, structure, and cross-linking degree (Figure 6).

The interstitial ECM of colorectal tumors is importantly characterized by an increase in fibrillar collagens, notably in collagen I, with thicker and more aligned fibers (Figure 6A) [161,162]. Along with increased collagen deposition, an overexpression of lysyl-oxidase (LOX) enhances collagen crosslinking, creating a dense and stiff microenvironment characteristic of tumor stroma fibrosis (Figure 6B) [163]. Interestingly, the mechanical stiffness of the TME has been shown to directly modulate immune cell recruitment and functions. For example, Kuczek et al. demonstrated that dense collagen matrices reduce proliferation and cytotoxic activities of T cells in vitro [164]. Similarly, it was observed in vivo that the fibrotic environment of CRC peritoneal metastases blocks T cells infiltration [165,166]. Conversely, while the stiff ECM may act as a physical barrier to T cells, dense collagen-rich ECM promotes macrophage infiltration and an immunosuppressive phenotype [167]. Apart from collagen I, collagen III is also upregulated CRC tumors [168].

In addition to collagens, many ECM glycoproteins, such as fibronectin or tenascin, have also been shown to be overexpressed in the tumor stroma, which modulate the biomechanical features of the TME and decorate it with multiple additional cell-adhesion sites. For example, fibronectin displays adhesive sites for the integrin β2 (CD18), which is widely expressed on leukocytes and plays an important role in their trafficking and during inflammatory responses [169]. Moreover, proteoglycans and glycosaminoglycans (GAGs) are significantly altered in CRC. For instance, hyaluronic acid (HA) is a glycosaminoglycan present in the interstitial and pericellular ECMs that participates in the hydration and mechanical properties of cells and tissues. Upon degradation, HA displays different immunomodulatory activities depending on the molecular size of the HA fragments [170,171]. Low-molecular-weight (LMW)-HA is known to have pro-inflammatory properties by promoting migration, activation and cytokine release of macrophages and T cells via CD44 signaling [170]. Interestingly, Zhang et al. recently showed that LMW-HA is preferentially increased in colorectal tumors, enhancing aggressiveness and metastasis [172].

Besides the interstitial ECM, alterations in the basement membranes (BMs) of tumors are known to affect intra- and extravasation of immune and tumor cells (Figure 6A). BMs are thin yet dense sheets of ECM proteins, mainly composed of collagen IV and laminin, which underly epithelia or surround blood and lymphatic vessels. In CRC, Spaderna et al. showed a local loss of BMs at the invasive front of the tumors, although most of BMs remain expressed in the tumor mass [173]. The loss of BMs results in a leaky vasculature with large pores and abnormal exposition of interstitial ECM components, such as fibrillar collagens. These differences in the matrix structure and biomechanical properties modulate cells mechanotransduction and trafficking [174,175]. For instance, it has been demonstrated that the differentiation of macrophages from monocytes is dependent on adhesion to BM components, particularly to laminins [176], suggesting that their loss might have substantial consequences on immune functions.

### 5.2. The ECM as a Reservoir of Immunomodulatory Proteins

Another important function of the ECM is its ability to interact with biomolecules and act as a reservoir. Biomolecules (e.g., cytokines, growth factors, proteases, EVs) can be locally secreted by cells or derived from the blood. By sequestering these biomolecules, the ECM tightly regulate their spatial and temporal release at the vicinity of the cells (Figure 6B) [159].

A well-known example is the ability of ECM to maintain and modulate chemokine gradients, which guide immune cells migration. These gradients can result from the interaction of chemokines with proteoglycans and GAGs. For example, the binding of CCL21 to heparan sulfate (HS) GAGs is necessary for gradient formation and subsequent directional migration of DCs [81,177]. Additionally, several other chemokines have been identified as displaying high affinities for GAGs, such as CXCL4, CXCL11, CXCL12γ, CCL5, all being important modulators of anti-tumor immunity [178].

In addition to binding to proteoglycans and GAGs, cytokines and chemokines also interact with ECM glycoproteins, which modulate their biological activities. For example, it has been shown in vitro that fibronectin-bound TNFα can arrest the migration of T cells along chemotactic gradients, acting as an anchoring signal [179]. In contrast to GAGs, fibronectin contains multiple integrin-binding sites, some located close to cytokine/growth factor-binding sites. Interestingly, this proximity of integrin- and growth factor-binding sites allows for synergistic signaling between the integrins and growth factors receptors, resulting in enhanced cellular responses [159,180]. While not being much explored in CRC, immune cell activities could likewise be modulated by differential signaling between ECM-bound and soluble cytokines or growth factors.

Another interesting example highlighting the ECM role in cytokine delivery is the molecular release mechanism of TGF-β [181]. TGF-β is secreted and stored in the ECM in a molecular “trap” called the Large Latent Complex (LLC), comprising a latency-associated peptide (LAP) and the latent TGF-β binding protein (LTBP). LTBP is an ECM glycoprotein incorporated in the matrix. The molecular trap remains closed until cell-surface integrins (e.g., αVβ6) bind to the LAP and exert a mechanical tension, which opens the trap and releases bioactive TGF-β. Upon release, TGF-β is rapidly captured by its receptors at the cell surface, preventing signaling on distant cells [182,183,184]. Interestingly, the stiffness of the ECM directly affects the mechanical force exerted by integrins on the LAP, such that stiff ECM permits TGF-β release, while soft ECM deforms without opening the trap. Therefore, ECM stiffening associated with CRC could substantially modulate the bioavailability of TGF-β to immune cells in the TME.

Finally, some cytokines or growth factors are released from the ECM upon proteolytic processing. For example, VEGF-C is secreted with a C-terminal propeptide that allows sequestration in the ECM [185]. To get fully active, VEGF-C propeptide has to be cleaved by proteases, notably by ADAMTS3 and plasmin. The mature VEGF-C then lacks ECM affinity, yet acquires high affinity for its receptor VEGF-R3. While in CRC, most studies focus on the role of VEGF-C in promoting tumor metastases [186], VEGF-C has also been shown to enhance immune cell trafficking and to increase macrophages and T cells recruitment in melanoma tumors [187,188,189].

### 5.3. Direct ECM Signaling to Immunoreceptors

Finally, ECM components or fragments can also be biologically active and directly signal to cell receptors. For example, osteopontin (OPN) is a matricellular protein which expression is correlated with poor prognosis in CRC. Interestingly, OPN modulates tumor immunosuppression by regulating myeloid cells and T cells, via the presence of a CD44-binding site [190]. Notably, Klement et al. highlighted that OPN suppresses proliferation, activation and IFNγ secretion by cytotoxic T cells through the OPN-CD44 signaling axis [191,192].

Additionally, ECM components can acquire signaling capability upon proteolysis, in which case the bioactive ECM fragment is referred as a matrikine. For example, the cleavage of cellular fibronectin by elastase-2 exposes its extra-domain A, which increases signaling via the toll-like receptor (TLR)-4 on immune cells [193]. In CRC, matrikines have been shown to have substantial effects on tumor inflammation. Particularly, Hope et al. have shown that versican-derived matrikines enhance CD8^+^ T cells infiltration by promoting DCs differentiation [194].

Lastly, some ECM components can directly activate the classical and alternative immune complement pathway. Indeed, fibromodulin (FMOD), which has been shown to be expressed in models of colon carcinoma [195], can activate the innate complement response by direct binding of the globular head of the C1q complement molecule [196]. However, ECM-complement interactions are mainly being studied in contexts other than cancer; therefore, their relevancy in tumor inflammation remains to be elucidated.

### 5.4. Indirect ECM-Mediated Immunomodulation via Anti-Microbial Activities

Lastly, an indirect immunomodulatory effect of the ECM is given by its anti-microbial function. Indeed, many ECM components contains highly positively charged or hydrophobic peptides that are able to permeate bacteria membranes and kill them [197]. ECM-derived anti-microbial peptides (AMPs) are often released upon ECM degradation during inflammation. The sensitivity of bacteria to specific AMPs depends on the composition of the bacteria cell wall. For example, *E. faecalis*, *E. coli* and *P. aeruginosa* are sensitive to the AMPs released from laminin, fibronectin and vitronectin [197]. In cancer, AMPs are often considered to be anti-tumor agents due to some direct cytotoxic actions on cancer cells or by enhancing tumor inflammation. While their roles in cancer are just emerging [198], one could hypothesize that AMPs affect the microbiome of colorectal tumors.

### 5.5. The Emergent Role of the Cell Glycocalyx in Cancer Immunity

Every cell is heavily coated with glycoproteins, GAGs, and glycosylated lipids. This cell coat is called glycocalyx and provides the cell with a pericellular ECM, which primarily keeps their hydration and dissipates surface shear stress (Figure 6C). Moreover, the components of the glycocalyx modulate mechanosignaling and the activity of soluble signaling molecules, similarly to the interstitial ECM [199]. The role of the glycocalyx in cancer remains emergent; however, multiple studies have shown that glycocalyces are dysregulated in cancer cells, modifying integrins functions and cell-surface receptor exposure [200,201,202]. Interestingly, the glycocalyx of cancer cells participates in immune evasion by shielding tumor cells from proper immune detection [203]. In addition to cancer cells, the glycocalyx of endothelial cells plays an important role in anti-tumor immunity. Indeed, the degradation of the endothelial glycocalyx favors the recruitment of immune cells by exposing selectins and integrins at the endothelium surface. This enables leukocytes to adhere and extravasate [204]. However, the function of the endothelial glycocalyx has been more explored during cancer cells extravasation and metastasis, rather than from the perspective of leukocyte infiltration [205,206].

In conclusion, while the ECM has been long considered as a passive cell scaffold, its multifaceted role in modulating inflammation in tumors has emerged. The ECM importantly regulates mechanosensing and biochemical signaling of immune cells in the TME. It additionally displays or releases bioactive pro- or anti-inflammatory peptides, as well as antimicrobial ones. Nevertheless, a lot remains to be understood about the crosstalk between ECM and tumor inflammation in CRC and in the different CMS.

## 6. Microbiome-Mediated Tumor Immunomodulation

Last but not least, the microbiome has recently been recognized as one of the hallmarks of cancer, due to its substantial effects on tumor development and immunity, and its presence in a variety of cancer [207,208]. In CRC particularly, the increased permeability of the gut barrier during cancer growth allows direct infiltration of the intestinal microbiota into the tumor. While the gut microbiota is known to contain bacteria, fungus, viruses or other microbes [209], current studies on the microbiome mainly focus on infiltrating bacteria. In this section, we highlight some mechanisms by which the microbiome modulates tumor inflammation, after briefly recalling the significance of the microbiome in the gut homeostasis and in CRC.

### 6.1. Intestinal Microbiota and Gut Immune Homeostasis

The gut commensal microbiota plays a pivotal role in the maintenance of host metabolism and in the defense against pathogen invasion. Importantly, the microbiome is essential for the development of a healthy colonic immune functions, hinting at an intricate crosstalk between the microbiota and local immune cell populations.

One vital function of a healthy microbiome is to establish and “train” a stable immune system, as particularly highlighted by studies in germ-free mice. Indeed, microbial depletion had detrimental effects on the development of both innate and adaptive immunity [210]. Germ-free mice are lacking vital mucosal immunity, are more susceptible to infections, and show signs of severe immuno-deficiency. They are characterized by dramatically reduced neutrophils counts, the lack of certain gut specific NK cells subsets, the downregulation of CD4^+^ T cells in the lamina propria and a reduced cytotoxic activity of intraepithelial CD8^+^ T cells and γδ T cells [211]. In addition to germ-free mice, manipulation of the microbiome by the introduction of new bacterial strains or antibiotic treatments have also been shown to disrupt gut immunity [212,213].

While the microbiome supports the development of gut immunity, many gut-localized immune cells are remarkably programmed to induce basal immune tolerance to a variety of microbes, thus allowing a symbiotic host-microbiota relationship [214]. Indeed, detection of homeostatic doses of microbial molecules by DCs and macrophages, for example, respectively results in high secretion of immunosuppressive IL-10 and in absence of pro-inflammatory cytokine production despite TLR stimulation [215]. This state of constant low level microbial stimulation of immune cells without pro-inflammatory responses is commonly referred as inflammation anergy. Additionally, the bacterial composition of the microbiome has been shown to regulate the polarization of CD4^+^ T cells into particular Th subsets. Notably, *Clostridia* and *Bacterioides fragilis* have been associated with the induction of colonic Tregs and the suppression of Th17 responses [216,217]. This regulation of CD4^+^ T cell polarization by the microbiome composition is essential in allowing rapid and effective pro-inflammatory responses in the case of pathological drift.

### 6.2. Pro- and Anti-Tumorigenic Microbial Inflammation

Persistent microbiome-associated inflammation is able to favor the onset of CRC, which has been highly correlated with the presence of specific microbial species in the gut. In addition, primary CRC tumors have been shown to be infiltrated with different bacteria strains, including the bacteroides *B. fragilis* and *B. dorei*, the fusobacterium *F. nucleatum*, the bacterium *Lachnospiraceae* [218], among others, generating diverse immunogenic effects in the TME. For example, the infiltration of *F. nucleatum* into colonic tumor lesions has been shown to support pro-tumorigenic inflammation via the recruitment of MDSCs and the modulation of T cell activity and NK cell cytotoxicity [219,220,221,222]. In addition, *F. nucleatum* activates the invariant TCR of mucosal-associated invariant T (MAIT) cells, present in CRC tumors, leading to an upregulation of PD-1 and CD39 expression and subsequent exhaustion [223].

In contrast, other bacterial species generate an anti-tumorigenic inflammatory environment. For instance, in patient biopsies, Cremonesi et al. showed that the presence of *Lachnospiraceae* and *Ruminococcaceaca* is correlated with increased T cell infiltration in the tumor, which associates with a more favorable patient prognosis [224]. In addition, the presence of *B. fragilis* in the ileum has been shown to promote accumulation and activation of follicular T helper cells (T_FH_) in CRC patients, leading to immunogenic cell death of IECs and higher efficacy of chemotherapy [225]. Together, these findings indicate that the microbiome composition in the gut and in the tumor have determinant roles on the type of tumor inflammation and subsequent growth.

### 6.3. Microbial Modulation of Tumor Inflammation

#### 6.3.1. PRR Activation

**Figure 7:**
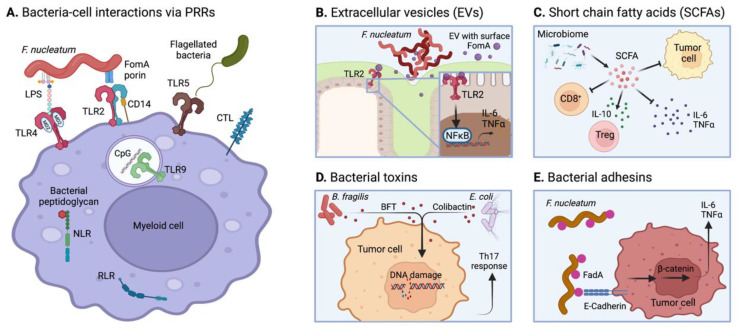
Examples of tumor immunomodulation by microbiome-cell interactions. (**A**) Activation of immune cells by PRR stimulation upon detection of bacterial-derived components. (**B**) Activation of PRR by bacterial-derived EVs and subsequent cytokine secretion. (**C**) Immunomodulation by SCFAs produced upon bacterial fermentation. (**D**) Indirect immunomodulation by bacterial toxins. (**E**) Induction of cytokine production upon bacteria adhesion on cell surface.

Microbial-associated molecular patterns (MAMPs), including the PAMPs, are primarily detected by PRR on myeloid cells to initiate inflammatory responses. PRRs are a superfamily of immunomodulatory receptors, which include the TLRs, the NOD-like receptors (NLRs), the C-type lectin receptors (CLRs) and the RIG-like receptors (RLRs), among others, together detecting a large variety of microbial-derived compounds (Figure 7A). For example, the FomA porins present on the membrane of the fusobacteria *F. nucleatum*, overrepresented in colorectal tumors [218], are detected by the TLR-2 and its co-receptor CD14 [226]. In addition, the high presence of lipopolysaccharides (LPS) on *F. nucleatum* stimulates TLR-4. Stimulation of TLR-2 and TLR-4 redundantly induces the secretion of the pro-inflammatory cytokines IL-6 and TNF-α from macrophages via the activation of NF-κB pathway [227]. In addition, TLR-5 detects bacteria flagellin and TLR-9 is activated by unmethylated Cytosine-phosphor-Guanine (CpG) motifs in bacterial DNA. On the other hand, activation of PRRs can rather result in immunosuppression. For instance, stimulation of NOD-1, which recognizes bacterial peptidoglycans-derived peptides, promotes immunosuppressive functions of MDSCs and macrophages as well as inhibits T cell responses, overall supporting tumor progression [228]. Other receptors such as lectins (e.g., Siglec-7) are also involved in bacterial glycans recognition and immunomodulation by myeloid cells [229]. In addition, detection of intratumoral bacteria by immune cells can trigger bacterial antigen presentation on MHC, bacterial phagocytosis, and secretion of anti-microbial compounds, among others classical anti-bacterial immune mechanisms. In the TME, the microbiome-associated immune response and tumor-induced inflammation likely take place jointly, one potentially affecting the other.

On the other hand, bacteria are also capable of detecting host molecules. Indeed, Abed et al. have demonstrated that fusobacteria homing into colorectal tumors is mediated by the recognition of Gal-Gal/NAC polysaccharides on tumor cells by the bacterial lectin Fap2 [219]. Therefore, bacteria not only interact with immune cells, but also with tumor cells.

#### 6.3.2. Microbial EVs

Similar to eukaryotic cells, microbes can shed portions of their membrane via EVs. Since many immunogenic microbial molecules are localized at their surface, EVs can be detected by PRRs to modulate immune cell responses. Indeed, the TLR-2 ligand FomA porin was detected on *F. nucleatum*-derived EVs and stimulates IECs to activate NFκB pathway, triggering the secretion of cytokines such as IL-8 (Figure 7B) [230].

#### 6.3.3. Short-Chain Fatty Acids (SCFAs)

One central metabolic role of the intestinal microbiome is the breakdown of indigestible polysaccharides, like dietary fibers and resistant starch, into SCFAs during bacterial fermentation. The main SCFAs are acetate, propionate, and butyrate. SCFAs have direct effects on CRC cells and on immune cells, respectively affecting tumor growth and inflammation. Indeed, they reduce proliferation of CRC cells and induce cell cycle arrest, cancer cell senescence and apoptosis, generally resulting in anti-tumor effects [231,232]. In parallel, SCFAs suppress pro-inflammatory cytokines production and rather induce secretion of anti-inflammatory cytokines (e.g., IL-10) by immune cells, thereby supporting the development of Treg and an immunosuppressive milieu in the gut (Figure 7C) [233,234]. Nevertheless, it has recently been shown that gut microbial-secreted butyrate improves anti-tumor efficacy of CD8+ T cells during chemotherapy, potentially highlighting a more complex immunomodulatory role of butyrate [235].

#### 6.3.4. Microbial Toxins

In addition, bacteria from the gut microbiome also produce toxins capable of inducing DNA damages in cells (referred as genotoxins) and of modulating immune responses (Figure 7D) [236]. For example, the *B. fragilis* toxin (BFT) and the *E. coli*-derived colibactin genotoxin have been associated with CRC onset and progression [237,238]. In addition, BFT has been shown to activate STAT3 pathway in IECs, inducing secretion of IL-17 and eliciting Th17-biased T cell responses in the gut [239]. Nevertheless, although these bacterial toxins are present in colorectal tumors [240], their effects on tumor inflammation remain poorly understood.

#### 6.3.5. Bacterial Adhesins

Lastly, adhesion molecules present on bacterial membranes can directly modulate the release of cytokines in the microenvironment by binding to cell-surface receptors. For example, FadA present on *F. nucleatum* promotes inflammation by interacting with E-cadherins on CRC cells, leading to the activation of β-catenin signaling and subsequent upregulation of pro-inflammatory IL-6 and TNF-α (Figure 7E) [241]. Similarly, some *E.coli* strains produce an afimbrial adhesin AFA-I to attach to IECs, and have been associated with CRC [242].

In conclusion, the gut microbiome is highly involved in the modulation of tumor inflammation during both CRC initiation and development. Importantly, modulating the composition of the gut microbiome via dietary conditions might represent a strong opportunity to prevent the development of CRC. In addition to the gut microbiome, the intratumoral microbiome can also regulate tumor inflammation by interacting with immune and other cells in the TME via a large diversity of bacterial ligands. Together, these interactions can result in pro- or anti-inflammatory mechanisms with positive or negative outcomes on tumor progression.

## 7. Conclusions: The Integrated Immunomodulatory Microenvironment of Colorectal Tumors and Therapeutic Perspectives

The goal of this review is to highlight key immunomodulatory molecular interactions taking place in tumors, with a particular focus on CRC. However, the significance of these interactions highly depends on the composition of the TME; for example, the microbiome is more represented in some tumor types, and some of the immune-tumor interactions might be inexistent in immune-excluded tumors.

In the TME, all immunomodulatory interactions are integrated; they occur in the same spatio-temporal frame, with one interaction dynamically influencing the other. Indeed, immune cells, tumor cells, stromal cells, soluble factors, the ECM and the microbiome interact together to shape tumor inflammation. In this complex interactive environment, cell behavior is dictated by the integration of all the signals received by a cell, and the net result of all cells’ behavior determines the tumor fate.

Taking advantage of tumor inflammation is widely explored as a strategy for the development of potent therapeutic. For example, ICI re-activates anti-cancer immune responses by relieving immune cells exhaustion, and BiTE activates immune cells by connecting them to tumor cells via molecular bridging. In theory, almost every immunomodulatory interaction could be targeted for the development of cancer immunotherapeutic, either inhibiting or activating it to modulate tumor inflammation. Nevertheless, predicting the efficacy of an immunotherapeutic drug according to the tumor composition and the disease stage remains an important challenge to overcome. Particularly, it would be important to define the responsiveness of CRC patients in function of their CMS-specific tumor types or oncogenic driving tumor mutations. In that perspective, systematic screening of tumor characteristics and improvement of diagnostic tools constitute essential advances.

On the other hand, it is likely that multiple immunomodulatory mechanisms would need to be targeted simultaneously or sequentially to prevent tumor immune escape. Indeed, combining immunotherapies with other cancer treatments that have been shown to modulate tumor inflammation (e.g., chemotherapy, targeted therapy, or other immunotherapy) provides an important option to potentiate treatments’ efficacy.

Currently, there are tremendous efforts focusing on the discovery, development, or improvement of immunomodulatory cancer drugs, as well as on predicting patient’s responsiveness, together providing high hopes for the treatment of mCRC in the coming years.

## Figures and Tables

**Table 1:** Examples of clinical trials using cytokine-based immunotherapy for colorectal cancer.

Immune Target	NTC Number	Phase	Title	Start Year
**Chemokines**				
CXCR1/2 inhibition	NCT04599140	1/2	SX-682 and Nivolumab for the Treatment of RAS-Mutated, MSS Unresectable or Metastatic Colorectal Cancer, the STOPTRAFFIC-1 Trial	2020
**CSFs**				
G-CSF	NCT00541125	2	Vaccine Therapy With or Without Sargramostim in Treating Patients With Advanced or Metastatic Cancer	2007
GM-CSF	NCT00028496	1	Vaccine Therapy in Treating Patients With Cancer of the Gastrointestinal Tract	2001
	NCT00012246	2	Vaccine Therapy in Treating Patients With Stage IIB, Stage III, or Stage IV Colorectal Cancer	2002
	NCT00091286	1	Cellular Immune Augmentation in Colon and Rectal Cancer	2003
	NCT00257322	2	Vaccine Therapy and Radiation to Liver Metastasis in Patients With CEA-Positive Solid Tumors	2003
	NCT00081848	1	Vaccine Therapy and Sargramostim With or Without Docetaxel in Treating Patients With Metastatic Lung Cancer or Metastatic Colorectal Cancer	2004
	NCT00088933	1	GM-CSF and Combination Chemotherapy in Treating Patients Who Are Undergoing Surgery for Stage II or Stage III Colon Cancer	2004
	NCT00262808	2	Vaccine Therapy in Treating Patients With Liver or Lung Metastases From Colorectal Cancer	2004
	NCT00103142	2	Study of Colon GVAX and Cyclophosphamide in Patients With Metastatic Colorectal Cancer	2005
	NCT00656123	1	Neoadjuvant Study of Recombinant Vaccinia Virus to Treat Metastatic Colorectal Carcinoma in Patients Undergoing Complete Resection of Liver Tumors	2008
	NCT01329809	2	Safety Study of Recombinant Vaccinia Virus Administered Intravenously in Patients With Metastatic, Refractory Colorectal Carcinoma	2011
	NCT01380600	1	SGI-110 in Combination With an Allogeneic Colon Cancer Cell Vaccine (GVAX) and Cyclophosphamide (CY) in Metastatic Colorectal Cancer (mCRC)	2011
	NCT01966289	1	RhGM-CSF as Adjuvant Immunotherapy in Treating Stage III Colon Cancer	2014
	NCT02466906	2	Study of GVAX (With CY) and Pembrolizumab in MMR-p Advanced Colorectal Cancer	2015
	NCT02981524	2	Vaccine Therapy With or Without Sargramostim in Treating Patients With Advanced or Metastatic Cancer	2017
**IFNs**				
IFN	NCT00309530	3	Randomized Study on Adjuvant Chemotherapy and Adjuvant Chemo-Immunotherapy in Colon Carcinoma Dukes C	1990
IFNα	NCT00003063	3	Biological Therapy With Combination Chemotherapy in Patients With Colorectal Cancer	1991
	NCT01060501	3	Modulation of Adjuvant 5-FU by Folinic Acid and Interferon-alpha in Colon Cancer	1992
	NCT02387307	1	A Study of rSIFN-co in Subjects With Advanced Solid Tumors	2013
	NCT04798612	2	Effect of Low-dose Interferon-alfa2a on Peri-operative Immune Suppression	2021
IFNα GM-CSF	NCT00016042	1	Fluorouracil and Biological Therapy in Treating Patients With Metastatic Kidney or Colorectal Cancer	2001
IFNα GM-CSF	NCT00030342	1/2	Biological Therapy and Chemotherapy in Treating Patients With Metastatic Kidney Cancer or Colorectal Cancer	2001
IFNα, IFNγ, GM-CSF	NCT00002475	2	Cyclophosphamide Plus Vaccine Therapy in Treating Patients With Advanced Cancer	1991
IFNγ	NCT00002796	1/2	Phase I-II Study of Fluorouracil in Combination With Phenylbutyrate in Advanced Colorectal Cancer	1997
**ILs**				
IL-2	NCT00019591	1/2	Vaccine Therapy With or Without Interleukin-2 in Treating Patients With Locally Advanced or Metastatic Colorectal Cancer	1999
	NCT00020267	1	Vaccine Therapy in Treating Patients With Metastatic Cancer	2000
	NCT03190941	1/2	Administering Peripheral Blood Lymphocytes Transduced With a Murine T-Cell Receptor Recognizing the G12V Variant of Mutated RAS in HLA-A*11:01 Patients	2017
	NCT03745326	1/2	Administering Peripheral Blood Lymphocytes Transduced With a Murine T-Cell Receptor Recognizing the G12D Variant of Mutated RAS in HLA-A*11:01 Patients	2019
	NCT04426669	1/2	A Study of Metastatic Gastrointestinal Cancers Treated With Tumor Infiltrating Lymphocytes in Which the Gene Encoding the Intracellular Immune Checkpoint CISH Is Inhibited Using CRISPR Genetic Engineering	2020
IL-2, GM-CSF	NCT00019331	2	Vaccine Therapy Plus Biological Therapy in Treating Adults With Metastatic Solid Tumors	1997
IL-2 fusion	NCT00128622	1	Denileukin Diftitox Followed by Vaccine Therapy in Treating Patients With Metastatic Cancer	2005
IL-2, GM-CSF	NCT00019084	2	Vaccine Therapy and Biological Therapy in Treating Patients With Advanced Cancer	1996
IL-7	NCT01339000	2	Improving the Immune System With Human IL-7 Vaccine in Older Subjects Who Have Had Chemotherapy	2011
IL-12	NCT00003046	1	Interleukin-12 in Treating Patients With Cancer in the Abdomen	1997
	NCT00003439	1	Interleukin-12 in Treating Patients With Refractory Advanced-Stage Ovarian Cancer or Abdominal Cancer	1998
	NCT00004074	1	Interleukin-12 and Trastuzumab in Treating Patients With Cancer That Has High Levels of HER2/Neu	1999
	NCT00072098	1	Interleukin-12 Gene in Treating Patients With Liver Metastases Secondary to Colorectal Cancer	2003
IL-15 super-agonist	NCT03127098	1/2	QUILT-3.040: ETBX-011 (Ad5 [E1-, E2b-]-CEA(6D)) Vaccine in Combination With ALT-803 (Super-agonist IL-15) in Subjects Having CEA-Expressing Cancer	2017
**TGF-β**				
TGF-β trap	NCT03436563	1/2	M7824 in Patients With Metastatic Colorectal Cancer or With Advanced Solid Tumors With Microsatellite Instability	2018
TGF-β trap IL-12 fusion	NCT04708470	1/2	Phase I/II Trial of the Combination of Bintrafusp Alfa (M7824), Entinostat and NHS-IL12 (M9241) in Patients With Advanced Cancer	2021
**TNFs**				
TNF	NCT00436410	1	Tumor Necrosis Factor in Patients Undergoing Surgery for Primary Cancer or Metastatic Cancer	2006
TNFα conjugate	NCT00098943	1	NGR-TNF in Treating Patients With Advanced Solid Tumors	2004

## Data Availability

Not applicable.

## References

[1] Henderson R.H., French D., Maughan T., Adams R., Allemani C., Minicozzi P., Coleman M.P., McFerran E., Sullivan R., Lawler M. (2021). The economic burden of colorectal cancer across Europe: A population-based cost-of-illness study. Lancet Gastroenterol. Hepatol..

[2] Holch J.W., Ricard I., Stintzing S., Modest D.P., Heinemann V. (2017). The relevance of primary tumour location in patients with metastatic colorectal cancer: A meta-analysis of first-line clinical trials. Eur. J. Cancer.

[3] André T., Shiu K.-K., Kim T.W., Jensen B.V., Jensen L.H., Punt C., Smith D., Garcia-Carbonero R., Benavides M., Gibbs P. (2020). Pembrolizumab in Microsatellite-Instability–High Advanced Colorectal Cancer. N. Engl. J. Med..

[4] Rumpold H., Niedersüß-Beke D., Heiler C., Falch D., Wundsam H.V., Metz-Gercek S., Piringer G., Thaler J. (2020). Prediction of mortality in metastatic colorectal cancer in a real-life population: A multicenter explorative analysis. BMC Cancer.

[5] Ganesh K., Stadler Z.K., Cercek A., Mendelsohn R.B., Shia J., Segal N.H., Diaz L.A. (2019). Immunotherapy in colorectal cancer: Rationale, challenges and potential. Nat. Rev. Gastroenterol. Hepatol..

[6] Kesselring R., Glaesner J., Hiergeist A., Naschberger E., Neumann H., Brunner S.M., Wege A.K., Seebauer C., Köhl G., Merkl S. (2016). IRAK-M Expression in Tumor Cells Supports Colorectal Cancer Progression through Reduction of Antimicrobial Defense and Stabilization of STAT3. Cell.

[7] Liu Y., Cao X. (2016). Characteristics and Significance of the Pre-metastatic Niche. Cancer Cell.

[8] Costa-Silva B., Aiello N.M., Ocean A.J., Singh S., Zhang H., Thakur B.K., Becker A., Hoshino A., Mark M.T., Molina H. (2015). Pancreatic cancer exosomes initiate pre-metastatic niche formation in the liver. Nat. Cell Biol..

[9] Erler T., Bennewith L., Cox R., Lang G., Bird D., Koong A., Le Q., Giaccia J. (2009). Hypoxia-induced lysis oxidase is a critical mediator of bone marrow cell recruitment to form the pre-metastatic niche. Cancer Cell.

[10] Hiratsuka S., Watanabe A., Sakurai Y., Akashi-Takamura S., Ishibashi S., Miyake K., Shibuya M., Akira S., Aburatani H., Maru Y. (2008). The S100A8–serum amyloid A3–TLR4 paracrine cascade establishes a pre-metastatic phase. Nat. Cell Biol..

[11] Hoshino A., Costa-Silva B., Shen T.-L., Rodrigues G., Hashimoto A., Tesic Mark M., Molina H., Kohsaka S., Di Giannatale A., Ceder S. (2015). Tumour exosome integrins determine organotropic metastasis. Nature.

[12] Seubert B., Grünwald B., Kobuch J., Cui H., Schelter F., Schaten S., Jams S., Lim N., Nagase H., Simonavicius N. (2015). TIMP-1 creates a pre-metastatic niche in the liver through SDF-1/ CXCR4-dependent neutrophil recruitment in mice. Hepatology.

[13] Dong Q., Liu X., Cheng K., Sheng J., Kong J., Liu T. (2021). Pre-metastatic Niche Formation in Different Organs Induced by Tumor Extracellular Vesicles. Front. Cell Dev. Biol..

[14] Hanahan D., Weinberg R. (2000). The Hallmarks of Cancer. Med. Lav..

[15] Fearon E., Vogelstein B. (1990). A Genetic Model for Colorectal Tumorigenesis. Cell.

[16] Brabletz T. (2012). To differentiate or not-routes towards metastasis. Nat. Rev. Cancer.

[17] Kamal Y., Schmit S.L., Frost H.R., Amos C.I. (2020). The tumor microenvironment of colorectal cancer metastases: Opportunities in cancer immunotherapy. Immunotherapy.

[18] Bertocchi A., Carloni S., Ravenda P.S., Bertalot G., Spadoni I., Lo Cascio A., Gandini S., Lizier M., Braga D., Asnicar F. (2021). Gut vascular barrier impairment leads to intestinal bacteria dissemination and colorectal cancer metastasis to liver. Cancer Cell.

[19] Barbirou M., Woldu H.G., Sghaier I., Bedoui S.A., Mokrani A., Aami R., Mezlini A., Yacoubi-Loueslati B., Tonellato P.J., Bouhaouala-Zahar B. (2020). Western influenced lifestyle and Kv2.1 association as predicted biomarkers for Tunisian colorectal cancer. BMC Cancer.

[20] Jin K., Ren C., Liu Y., Lan H., Wang Z. (2020). An update on colorectal cancer microenvironment, epigenetic and immunotherapy. Int. Immunopharmacol..

[21] Frigerio S., Lartey D.A., D’haens G.R., Grootjans J. (2021). The role of the immune system in ibd-associated colorectal cancer: From pro to anti-tumorigenic mechanisms. Int. J. Mol. Sci..

[22] Guinney J., Dienstmann R., Wang X., De Reyniès A., Schlicker A., Soneson C., Marisa L., Roepman P., Nyamundanda G., Angelino P. (2015). The consensus molecular subtypes of colorectal cancer. Nat. Med..

[23] Picard E., Verschoor C.P., Ma G.W., Pawelec G. (2020). Relationships Between Immune Landscapes, Genetic Subtypes and Responses to Immunotherapy in Colorectal Cancer. Front. Immunol..

[24] Fabian K.P., Wolfson B., Hodge J.W. (2021). From Immunogenic Cell Death to Immunogenic Modulation: Select Chemotherapy Regimens Induce a Spectrum of Immune-Enhancing Activities in the Tumor Microenvironment. Front. Oncol..

[25] You Q., Fang T., Yin X., Wang Y., Yang Y., Zhang L., Xue Y. (2021). Serum CD4 Is Associated with the Infiltration of CD4+T Cells in the Tumor Microenvironment of Gastric Cancer. J. Immunol. Res..

[26] Eissner G., Kirchner S., Lindner H., Kolch W., Janosch P., Grell M., Scheurich P., Andreesen R., Holler E. (2000). Reverse Signaling Through Transmembrane TNF Confers Resistance to Lipopolysaccharide in Human Monocytes and Macrophages. J. Immunol..

[27] Briquez P.S., Hauert S., de Titta A., Gray L.T., Alpar A.T., Swartz M.A., Hubbell J.A. (2020). Engineering Targeting Materials for Therapeutic Cancer Vaccines. Front. Bioeng. Biotechnol..

[28] Wagner S., Mullins C.S., Linnebacher M. (2018). Colorectal cancer vaccines: Tumor-associated antigens vs neoantigens. World J. Gastroenterol..

[29] Li C., Liu T., Yin L., Zuo D., Lin Y., Wang L. (2019). Prognostic and clinicopathological value of MUC1 expression in colorectal cancer; A meta-analysis. Medicine.

[30] Simons K.H., de Jong A., Jukema J.W., de Vries M.R., Arens R., Quax P.H.A. (2019). T cell co-stimulation and co-inhibition in cardiovascular disease: A double-edged sword. Nat. Rev. Cardiol..

[31] Chen L., Flies D.B. (2013). Molecular mechanisms of T cell co-stimulation and co-inhibition. Nat. Rev. Immunol..

[32] Llosa N.J., Cruise M., Tam A., Wicks E.C., Hechenbleikner E.M., Taube J.M., Blosser R.L., Fan H., Wang H., Luber B.S. (2015). The vigorous immune microenvironment of microsatellite instable colon cancer is balanced by multiple counter-inhibitory checkpoints. Cancer Discov..

[33] Munn D.H., Bronte V. (2016). Immune suppressive mechanisms in the tumor microenvironment. Curr. Opin. Immunol..

[34] Sznol M., Chen L. (2013). Antagonist antibodies to PD-1 and B7-H1 (PD-L1) in the treatment of advanced human cancer. Clin. Cancer Res..

[35] Oyewole-Said D., Konduri V., Vazquez-Perez J., Weldon S.A., Levitt J.M., Decker W.K. (2020). Beyond T-Cells: Functional Characterization of CTLA-4 Expression in Immune and Non-Immune Cell Types. Front. Immunol..

[36] Gao Y., Wu Y., Du J., Zhan Y., Sun D., Zhao J., Zhang S., Li J., He K. (2017). Both light-induced SA accumulation and ETI mediators contribute to the cell death regulated by BAK1 and BKK1. Front. Plant Sci..

[37] de Miguel D., Lemke J., Anel A., Walczak H., Martinez-Lostao L. (2016). Onto better TRAILs for cancer treatment. Cell Death Differ..

[38] Houston J.P. (2019). Apoptosis and autophagy. Cytom. Part A.

[39] Huber V., Fais S., Iero M., Lugini L., Canese P., Squarcina P., Zaccheddu A., Colone M., Arancia G., Gentile M. (2005). Human colorectal cancer cells induce T-cell death through release of proapoptotic microvesicles: Role in immune escape. Gastroenterology.

[40] Yoon S.R., Kim T.D., Choi I. (2015). Understanding of molecular mechanisms in natural killer cell therapy. Exp. Mol. Med..

[41] McGilvray R.W., Eagle R.A., Watson N.F.S., Al-Attar A., Ball G., Jafferji I., Trowsdale J., Durrant L.G. (2009). NKG2D ligand expression in human colorectal cancer reveals associations with prognosis and evidence for immunoediting. Clin. Cancer Res..

[42] Chao M.P., Takimoto C.H., Feng D.D., McKenna K., Gip P., Liu J., Volkmer J.P., Weissman I.L., Majeti R. (2020). Therapeutic Targeting of the Macrophage Immune Checkpoint CD47 in Myeloid Malignancies. Front. Oncol..

[43] Banerjea A., Ahmed S., Hands R.E., Huang F., Han X., Shaw P.M., Feakins R., Bustin S.A., Dorudi S. (2004). Colorectal cancers with microsatellite instability display mRNA expression signatures characteristic of increased immunogenicity. Mol. Cancer.

[44] Fujiwara-Tani R., Sasaki T., Ohmori H., Luo Y., Goto K., Nishiguchi Y., Mori S., Nakashima C., Mori T., Miyagawa Y. (2019). Concurrent Expression of CD47 and CD44 in Colorectal Cancer Promotes Malignancy. Pathobiology.

[45] Mohme M., Riethdorf S., Pantel K. (2017). Circulating and disseminated tumour cells-mechanisms of immune surveillance and escape. Nat. Rev. Clin. Oncol..

[46] Barkal A.A., Weiskopf K., Kao K.S., Gordon S.R., Rosental B., Yiu Y.Y., George B.M., Markovic M., Ring N.G., Tsai J.M. (2018). Engagement of MHC class i by the inhibitory receptor LILRB1 suppresses macrophages and is a target of cancer immunotherapy article. Nat. Immunol..

[47] Barkal A.A., Brewer R.E., Markovic M., Kowarsky M., Barkal S.A., Zaro B.W., Krishnan V., Hatakeyama J., Dorigo O., Barkal L.J. (2019). CD24 signalling through macrophage Siglec-10 is a target for cancer immunotherapy. Nature.

[48] Feng K., Guo Y., Dai H., Wang Y., Li X., Jia H., Han W. (2016). Chimeric antigen receptor-modified T cells for the immunotherapy of patients with EGFR-expressing advanced relapsed/refractory non-small cell lung cancer. Sci. China Life Sci..

[49] Shin J.H., Jeong J., Maher S.E., Lee H.-W., Lim J., Bothwell A.L.M. (2021). Colon cancer cells acquire immune regulatory molecules from tumor-infiltrating lymphocytes by trogocytosis. Proc. Natl. Acad. Sci. USA.

[50] Sabzevari H., Kantor J., Jaigirdar A., Tagaya Y., Naramura M., Hodge J.W., Bernon J., Schlom J. (2001). Acquisition of CD80 (B7-1) by T Cells. J. Immunol..

[51] Dopfer E.P., Minguet S., Schamel W.W.A. (2011). A New Vampire Saga: The Molecular Mechanism of T Cell Trogocytosis. Immunity.

[52] Schnoor M., Lai F.P.L., Zarbock A., Kläver R., Polaschegg C., Schulte D., Weich H.A., Oelkers J.M., Rottner K., Vestweber D. (2011). Cortactin deficiency is associated with reduced neutrophil recruitment but increased vascular permeability in vivo. J. Exp. Med..

[53] Strell C., Paulsson J., Jin S.B., Tobin N.P., Mezheyeuski A., Roswall P., Mutgan C., Mitsios N., Johansson H., Wickberg S.M. (2019). Impact of Epithelial-Stromal Interactions on Peritumoral Fibroblasts in Ductal Carcinoma in Situ. J. Natl. Cancer Inst..

[54] Thomas S.N., Zhu F., Schnaar R.L., Alves C.S., Konstantopoulos K. (2008). Carcinoembryonic antigen and CD44 variant isoforms cooperate to mediate colon carcinoma cell adhesion to E- and L-selectin in shear flow. J. Biol. Chem..

[55] Wang W., Erbe A.K., Hank J.A., Morris Z.S., Sondel P.M. (2015). NK cell-mediated antibody-dependent cellular cytotoxicity in cancer immunotherapy. Front. Immunol..

[56] Gül N., van Egmond M. (2015). Antibody-dependent phagocytosis of tumor cells by Macrophages: A Potent effector mechanism of monoclonal antibody therapy of cancer. Cancer Res..

[57] Seo Y., Ishii Y., Ochiai H., Fukuda K., Akimoto S., Hayashida T., Okabayashi K., Tsuruta M., Hasegawa H., Kitagawa Y. (2014). Cetuximab-mediated ADCC activity is correlated with the cell surface expression level of EGFR but not with the KRAS/BRAF mutational status in colorectal cancer. Oncol. Rep..

[58] Veluchamy J.P., Spanholtz J., Tordoir M., Thijssen V.L., Heideman D.A.M., Verheul H.M.W., De Gruijl T.D., Van Der Vliet H.J. (2016). Combination of NK cells and cetuximab to enhance anti-tumor responses in RAS mutant metastatic colorectal cancer. PLoS ONE.

[59] Gerdes C.A., Nicolini V.G., Herter S., Van Puijenbroek E., Lang S., Roemmele M., Moessner E., Freytag O., Friess T., Ries C.H. (2013). GA201 (RG7160): A novel, humanized, glycoengineered anti—EGFR antibody with enhanced ADCC and superior in vivo efficacy compared with cetuximab. Clin. Cancer Res..

[60] Lemke G. (2017). Phosphatidylserine is the signal for TAM receptors and their ligands. Trends Biochem. Sci..

[61] Lemke G., Rothlin C.V. (2008). Immunobiology of the TAM receptors. Nat. Rev. Immunol..

[62] Werfel A., Cook S. (2016). Efferocytosis in the Tumor Microenvironment. Encycl. Cell Biol..

[63] Akitake-Kawano R., Seno H., Nakatsuji M., Kimura Y., Nakanishi Y., Yoshioka T., Kanda K., Kawada M., Kawada K., Sakai Y. (2013). Inhibitory role of Gas6 in intestinal tumorigenesis. Carcinogenesis.

[64] Jia M., Yao H., Chen C., Wang Y., Wang H., Cui T., Zhu J. (2017). Prognostic Correlation Between MFG-E8 Expression Level and Colorectal Cancer. Arch. Med. Res..

[65] Zhou Y., Yao Y., Deng Y., Shao A. (2020). Regulation of efferocytosis as a novel cancer therapy. Cell Commun. Signal..

[66] Josephs S.F., Ichim T.E., Prince S.M., Kesari S., Marincola F.M., Escobedo A.R., Jafri A. (2018). Unleashing endogenous TNF-alpha as a cancer immunotherapeutic. J. Transl. Med..

[67] Ardestani S., Deskins D.L., Young P.P. (2013). Membrane TNF-alpha-activated programmed necrosis is mediated by Ceramide-induced reactive oxygen species. J. Mol. Signal..

[68] Crocker P.R., Paulson J.C., Varki A. (2007). Siglecs and their roles in the immune system. Nat. Rev. Immunol..

[69] Yu Y., Blokhuis B.R.J., Diks M.A.P., Keshavarzian A., Garssen J., Redegeld F.A. (2018). Functional inhibitory siglec-6 is upregulated in human colorectal cancer-associated mast cells. Front. Immunol..

[70] Pasquale E.B. (2019). Eph receptors and ephrins engage in cellular cannibalism. J. Cell Biol..

[71] Aasen T., Mesnil M., Naus C.C., Lampe P.D., Laird D.W. (2016). Gap junctions and cancer: Communicating for 50 years. Nat. Rev. Cancer.

[72] Bristol Myers Squibb (2018). Opdivo (Nivolumab) in Combination with Yervoy (Ipilimumab) Demonstrates Clinical Activity in Previously Treated Patients with dMMR or MSI-H Metastatic Colorectal Cancer.

[73] Dinarello C.A. (2007). Historical Review of Cytokines. Eur. J. Immunol..

[74] Nagarsheth N., Wicha M.S., Zou W. (2017). Chemokines in the cancer microenvironment and their relevance in cancer immunotherapy. Nat. Rev. Immunol..

[75] Balkwill F. (2004). Cancer and the chemokine network. Nat. Rev. Cancer.

[76] Zlotnik A., Yoshie O. (2011). The Chemokine Superfamily Revisited NIH Public Access. Bone.

[77] Franca-Koh J., Kamimura Y., Devreotes P. (2006). Navigating signaling networks: Chemotaxis in Dictyostelium discoideum. Curr. Opin. Genet. Dev..

[78] Jin T., Xu X., Hereld D. (2008). Chemotaxis, chemokine receptors and human disease. Cytokine.

[79] Kohli K., Pillarisetty V.G., Kim T.S. (2021). Key chemokines direct migration of immune cells in solid tumors. Cancer Gene Ther..

[80] Karin N., Wildbaum G. (2015). The role of chemokines in shaping the balance between CD4+ T cell subsets and its therapeutic implications in autoimmune and cancer diseases. Front. Immunol..

[81] Weber M., Hauschild R., Schwarz J., Moussion C., De Vries I., Legler D.F., Luther S.A., Bollenbach T., Sixt M. (2013). Interstitial dendritic cell guidance by haptotactic chemokine gradients. Science.

[82] Günther K., Leier J., Henning G., Dimmler A., Weißbach R., Hohenberger W., Förster R. (2005). Prediction of lymph node metastasis in colorectal carcinoma by expression of chemokine receptor CCR7. Int. J. Cancer.

[83] Mantovani A., Savino B., Locati M., Zammataro L., Allavena P., Bonecchi R. (2010). The chemokine system in cancer biology and therapy. Cytokine Growth Factor Rev..

[84] Brocker C., Thompson D., Matsumoto A., Nebert D.W., Vasiliou V. (2010). Evolutionary divergence and functions of the human interleukin (IL) gene family. Hum. Genom..

[85] Briukhovetska D., Dörr J., Endres S., Libby P., Dinarello C.A., Kobold S. (2021). Interleukins in cancer: From biology to therapy. Nat. Rev. Cancer.

[86] Li J., Huang L., Zhao H., Yan Y., Lu J. (2020). The role of interleukins in colorectal cancer. Int. J. Biol. Sci..

[87] Akdis M., Aab A., Altunbulakli C., Azkur K., Costa R.A., Crameri R., Duan S., Eiwegger T., Eljaszewicz A., Ferstl R. (2016). Interleukins (from IL-1 to IL-38), interferons, transforming growth factor β, and TNF-α: Receptors, functions, and roles in diseases. J. Allergy Clin. Immunol..

[88] Spolski R., Li P., Leonard W.J. (2018). Biology and regulation of IL-2: From molecular mechanisms to human therapy. Nat. Rev. Immunol..

[89] Cooper A.M., Magram J., Ferrante J., Orme I.M. (1997). Interleukin 12 (IL-12) is crucial to the development of protective immunity in mice intravenously infected with mycobacterium tuberculosis. J. Exp. Med..

[90] Tosolini M., Kirilovsky A., Mlecnik B., Fredriksen T., Mauger S., Bindea G., Berger A., Bruneval P., Fridman W.H., Pagès F. (2011). Clinical impact of different classes of infiltrating T cytotoxic and helper cells (Th1, Th2, Treg, Th17) in patients with colorectal cancer. Cancer Res..

[91] Nguyen K.G., Vrabel M.R., Mantooth S.M., Hopkins J.J., Wagner E.S., Gabaldon T.A., Zaharoff D.A. (2020). Localized Interleukin-12 for Cancer Immunotherapy. Front. Immunol..

[92] Lin X., Wang S., Sun M., Zhang C., Wei C., Yang C., Dou R., Liu Q., Xiong B. (2019). MiR-195-5p/NOTCH2-mediated EMT modulates IL-4 secretion in colorectal cancer to affect M2-like TAM polarization. J. Hematol. Oncol..

[93] Junttila I.S., Mizukami K., Dickensheets H., Meier-Schellersheim M., Yamane H., Donnelly R.P., Paul W.E. (2008). Tuning sensitivity to IL-4 and IL-13: Differential expression of IL-4Rα, IL-13Ra1, and γc regulates relative cytokine sensitivity. J. Exp. Med..

[94] Gamez-Belmonte R., Erkert L., Wirtz S., Becker C. (2020). The regulation of intestinal inflammation and cancer development by type 2 immune responses. Int. J. Mol. Sci..

[95] De Simone V., Franzè E., Ronchetti G., Colantoni A., Fantini M.C., Di Fusco D., Sica G.S., Sileri P., MacDonald T.T., Pallone F. (2015). Th17-type cytokines, IL-6 and TNF-α synergistically activate STAT3 and NF-kB to promote colorectal cancer cell growth. Oncogene.

[96] Hernandez C., Huebener P., Schwabe R.F. (2016). Damage-associated molecular patterns in cancer: A double-edged sword. Oncogene.

[97] Deng T., Tang C., Zhang G., Wan X. (2021). DAMPs released by pyroptotic cells as major contributors and therapeutic targets for CAR-T-related toxicities. Cell Death Dis..

[98] Ivashkiv B., Donlin T. (2014). Regulation of type I interferon responses. Nat. Rev. Immunol..

[99] Walter M.R. (2020). The Role of Structure in the Biology of Interferon Signaling. Front. Immunol..

[100] Zitvogel L., Galluzzi L., Kepp O., Smyth M.J., Kroemer G. (2015). Type I interferons in anticancer immunity. Nat. Rev. Immunol..

[101] Budhwani M., Mazzieri R., Dolcetti R. (2018). Plasticity of type I interferon-mediated responses in cancer therapy: From anti-tumor immunity to resistance. Front. Oncol..

[102] Johdi N.A., Sukor N.F. (2020). Colorectal Cancer Immunotherapy: Options and Strategies. Front. Immunol..

[103] Castro F., Cardoso A.P., Gonçalves R.M., Serre K., Oliveira M.J. (2018). Interferon-gamma at the crossroads of tumor immune surveillance or evasion. Front. Immunol..

[104] Cooper M.A., Elliott J.M., Keyel P.A., Yang L., Carrero J.A., Yokoyama W.M. (2009). Cytokine-induced memory-like natural killer cells. Proc. Natl. Acad. Sci. USA.

[105] Chen B., Alvarado D.M., Iticovici M., Kau N.S., Park H., Parikh P.J., Thotala D., Ciorba M.A. (2020). Therapeutic Target in Colorectal Cancer. World J. Gastrointest. Oncol..

[106] Croft M., Benedict C.A., Ware C.F. (2013). Clinical targeting of the TNF and TNFR superfamilies. Nat. Rev. Drug Discov..

[107] Lee W.H., Seo D., Lim S.G., Suk K. (2019). Reverse Signaling of Tumor Necrosis Factor Superfamily Proteins in Factor Superfamily Proteins in Macrophages and microgia: Superfamily portrait in the neuroimmune interface. Front. Immunol..

[108] Bremer E. (2013). Targeting of the Tumor Necrosis Factor Receptor Superfamily for Cancer Immunotherapy. ISRN Oncol..

[109] Balkwill F. (2006). TNF-α in promotion and progression of cancer. Cancer Metastasis Rev..

[110] Montfort A., Colacios C., Levade T., Andrieu-Abadie N., Meyer N., Ségui B. (2019). The TNF paradox in cancer progression and immunotherapy. Front. Immunol..

[111] Zhao X., Rong L., Zhao X., Li X., Liu X., Deng J., Wu H., Xu X., Erben U., Wu P. (2012). TNF signaling drives myeloid-derived suppressor cell accumulation. J. Clin. Investig..

[112] Al Obeed O.A., Alkhayal K.A., Al Sheikh A., Zubaidi A.M., Vaali-Mohammed M.A., Boushey R., Mckerrow J.H., Abdulla M.H. (2014). Increased expression of tumor necrosis factor-α is associated with advanced colorectal cancer stages. World J. Gastroenterol..

[113] Metcalf D., Nicola N.A. (1983). Proliferative effects of purified granulocyte colony-stimulating factor (G-CSF) on normal mouse hemopoietic cells. J. Cell. Physiol..

[114] Hamilton J.A. (2019). GM-CSF in inflammation. J. Exp. Med..

[115] Shi Y., Liu C.H., Roberts A.I., Das J., Xu G., Ren G., Zhang Y., Zhang L., Zeng R.Y., Tan H.S.W. (2006). Granulocyte-macrophage colony-stimulating factor (GM-CSF) and T-cell responses: What we do and don’t know. Cell Res..

[116] Nebiker C.A., Han J., Eppenberger-Castori S., Iezzi G., Hirt C., Amicarella F., Cremonesi E., Huber X., Padovan E., Angrisani B. (2014). GM-CSF production by tumor cells is associated with improved survival in colorectal cancer. Clin. Cancer Res..

[117] Chen G., Han G., Shen B., Li Y. (2014). GM-CSF facilitates the development of inflammation-associated colorectal carcinoma. Oncoimmunology.

[118] Wakefield L.M., Kondaiah P., Hollands R.S., Winokur T.S., Sporn M.B. (1991). Addition of a C-Terminal Extension Sequence to Transforming Growth Factor-pl Interferes with Biosynthetic Processing and Abolishes Biological Activity. Growth Factors.

[119] Kelly A., Gunaltay S., McEntee C.P., Shuttleworth E.E., Smedley C., Houston S.A., Fenton T.M., Levison S., Mann E.R., Travis M.A. (2018). Human monocytes and macrophages regulate immune tolerance via integrin αvβ8-mediated TGFβ activation. J. Exp. Med..

[120] Tacconi C., Ungaro F., Correale C., Arena V., Massimino L., Detmar M., Spinelli A., Carvello M., Mazzone M., Oliveira A.I. (2019). Activation of the VEGFC/VEGFR3 pathway induces tumor immune escape in colorectal cancer. Cancer Res..

[121] Clements V.K., Long T., Long R., Figley C., Smith D.M.C., Ostrand-Rosenberg S. (2018). Frontline Science: High fat diet and leptin promote tumor progression by inducing myeloid-derived suppressor cells. J. Leukoc. Biol..

[122] Abolhassani M., Aloulou N., Chaumette M.T., Aparicio T., Martin-Garcia N., Mansour H., Le Gouvello S., Delchier J.C., Sobhani I. (2008). Leptin receptor-related immune response in colorectal tumors: The role of colonocytes and interleukin-8. Cancer Res..

[123] López-Otín C., Bond J.S. (2008). Proteases: Multifunctional enzymes in life and disease. J. Biol. Chem..

[124] Afonina I.S., Müller C., Martin S.J., Beyaert R. (2015). Proteolytic Processing of Interleukin-1 Family Cytokines: Variations on a Common Theme. Immunity.

[125] Pyrillou K., Burzynski L.C., Clarke M.C.H. (2020). Alternative Pathways of IL-1 Activation, and Its Role in Health and Disease. Front. Immunol..

[126] Zhao W., Oskeritzian C.A., Pozez A.L., Schwartz L.B. (2005). Cytokine Production by Skin-Derived Mast Cells: Endogenous Proteases Are Responsible for Degradation of Cytokines. J. Immunol..

[127] Shields J.D., Fleury M.E., Yong C., Tomei A.A., Randolph G.J., Swartz M.A. (2007). Autologous Chemotaxis as a Mechanism of Tumor Cell Homing to Lymphatics via Interstitial Flow and Autocrine CCR7 Signaling. Cancer Cell.

[128] Fleury M.E., Boardman K.C., Swartz M.A. (2006). Autologous morphogen gradients by subtle interstitial flow and matrix interactions. Biophys. J..

[129] Schumann K., Lämmermann T., Bruckner M., Legler D.F., Polleux J., Spatz J.P., Schuler G., Förster R., Lutz M.B., Sorokin L. (2010). Immobilized Chemokine Fields and Soluble Chemokine Gradients Cooperatively Shape Migration Patterns of Dendritic Cells. Immun..

[130] Lorenz N., Loef E.J., Kelch I.D., Verdon D.J., Black M.M., Middleditch M.J., Greenwood D.R., Graham E.S., Brooks A.E.S., Dunbar P.R. (2016). Plasmin and regulators of plasmin activity control the migratory capacity and adhesion of human T cells and dendritic cells by regulating cleavage of the chemokine CCL21. Immunol. Cell Biol..

[131] Said A.H., Raufman J.P., Xie G. (2014). The role of matrix metalloproteinases in colorectal cancer. Cancers.

[132] López-Otín C., Matrisian L.M. (2007). Emerging roles of porteases in tumour suppression. Tumour Microenviron. Opin..

[133] Jackson H.W., Defamie V., Waterhouse P., Khokha R. (2017). TIMPs: Versatile extracellular regulators in cancer. Nat. Rev. Cancer.

[134] Shay G., Lynch C.C., Fingleton B. (2015). Moving targets: Emerging roles for MMPs in cancer progression and metastasis. Matrix Biol..

[135] Kirkin V., Joos S., Zörnig M. (2004). The role of Bcl-2 family members in tumorigenesis. Biochim. Biophys. Acta Mol. Cell Res..

[136] Schulte M., Reiss K., Lettau M., Maretzky T., Ludwig A., Hartmann D., de Strooper B., Janssen O., Saftig P. (2007). ADAM10 regulates FasL cell surface expression and modulates FasL-induced cytotoxicity and activation-induced cell death. Cell Death Differ..

[137] Song E., Chen J., Ouyang N., Su F., Wang M., Heemann U. (2001). Soluble Fas ligand released by colon adenocarcinoma cells induces host lymphocyte apoptosis: An active mode of immune evasion in colon cancer. Br. J. Cancer.

[138] Barros F.M., Carneiro F., Machado J.C., Melo S.A. (2018). Exosomes and immune response in cancer: Friends or foes?. Front. Immunol..

[139] Shah R., Patel T., Freedman E. (2018). Circulating Extracellular Vesicles in Human Disease. N. Engl. J. Med..

[140] Mannavola F., Salerno T., Passarelli A., Tucci M., Internò V., Silvestris F. (2019). Revisiting the role of exosomes in colorectal cancer: Where are we now?. Front. Oncol..

[141] Mimoto F., Tatsumi K., Shimizu S., Kadono S., Haraya K., Nagayasu M., Suzuki Y., Fujii E., Kamimura M., Hayasaka A. (2020). Exploitation of Elevated Extracellular ATP to Specifically Direct Antibody to Tumor Microenvironment. Cell Rep..

[142] Ohta A., Gorelik E., Prasad S.J., Ronchese F., Lukashev D., Wong M.K.K., Huang X., Caldwell S., Liu K., Smith P. (2006). A2A adenosine receptor protects tumors from antitumor T cells. Proc. Natl. Acad. Sci. USA.

[143] Hajizadeh F., Masjedi A., Heydarzedeh Asl S., Karoon Kiani F., Peydaveisi M., Ghalamfarsa G., Jadidi-Niaragh F., Sevbitov A. (2020). Adenosine and adenosine receptors in colorectal cancer. Int. Immunopharmacol..

[144] Li X., Nie J., Mei Q., Han W.D. (2016). MicroRNAs: Novel immunotherapeutic targets in colorectal carcinoma. World J. Gastroenterol..

[145] Shao Y., Chen T., Zheng X., Yang S., Xu K., Chen X., Xu F., Wang L., Shen Y., Wang T. (2018). Colorectal Cancer-derived Small Extracellular Cesicles Establish an Inflammatory Pre-metastatic Niche in Liver Metastasisi. Carcinogenesis.

[146] Masucci M.T., Minopoli M., del Vecchio S., Carriero M.V. (2020). The Emerging Role of Neutrophil Extracellular Traps (NETs) in Tumor Progression and Metastasis. Front. Immunol..

[147] SenGupta S., Subramnian C., Parent A. (2019). Getting TANned: How the tumor microenvironment drive neutrophil recruitment. J. Leukoc. Biol..

[148] Teijeira Á., Garasa S., Gato M., Alfaro C., Migueliz I., Cirella A., de Andrea C., Ochoa M.C., Otano I., Etxeberria I. (2020). CXCR1 and CXCR2 Chemokine Receptor Agonists Produced by Tumors Induce Neutrophil Extracellular Traps that Interfere with Immune Cytotoxicity. Immunity.

[149] Khan M., Arooj S., Wang H. (2020). NK Cell-Based Immune Checkpoint Inhibition. Front. Immunol..

[150] Timosenko E., Hadjinicolaou V., Cerundolo V. (2017). Modulation of cancer-specific immune responses by amino acid degrading enzymes. Immunotherapy.

[151] Grzywa T.M., Sosnowska A., Matryba P., Rydzynska Z., Jasinski M., Nowis D., Golab J. (2020). Myeloid Cell-Derived Arginase in Cancer Immune Response. Front. Immunol..

[152] Munn D.H. (2012). Indoleamine 2,3-dioxygenase, Tregs and Cancer. Curr. Med. Chem..

[153] Benedetti S., Nuvoli B., Catalani S., Galati R. (2015). Reactive oxygen species a double-edged sword for mesothelioma. Oncotarget.

[154] Perillo B., Di Donato M., Pezone A., Di Zazzo E., Giovannelli P., Galasso G., Castoria G., Migliaccio A. (2020). ROS in cancer therapy: The bright side of the moon. Exp. Mol. Med..

[155] Kotsafti A., Scarpa M., Castagliuolo I., Scarpa M. (2020). Reactive oxygen species and antitumor immunity—from surveillance to evasion. Cancers.

[156] Chen L., Tseng H., Chen Y., Tanzih A., Haq A., Hwang P., Hsu H. (2020). Oligo-Fucoidan Prevents M2 Macrophage Differentiation and HCT116 Tumor Progression. Cancer.

[157] Finetti F., Travelli C., Ercoli J., Colombo G., Buoso E., Trabalzini L. (2020). Prostaglandin E2 and cancer: Insight into tumor progression and immunity. Biology.

[158] Nardy A.F.F.R., Freire-de-Lima L., Freire-de-Lima C.G., Morrot A. (2016). The sweet side of immune evasion: Role of Glycans in the Mechanisms of Cancer Progression. Front. Oncol..

[159] Briquez P.S., Hubbell J.A., Martino M.M. (2015). Extracellular Matrix-Inspired Growth Factor Delivery Systems for Skin Wound Healing. Adv. Wound Care.

[160] Winkler J., Abisoye-Ogunniyan A., Metcalf K.J., Werb Z. (2020). Concepts of extracellular matrix remodelling in tumour progression and metastasis. Nat. Commun..

[161] Birk J.W., Tadros M., Moezardalan K., Nadyarnykh O., Forouhar F., Anderson J., Campagnola P. (2014). Second harmonic generation imaging distinguishes both high-grade dysplasia and cancer from normal colonic mucosa. Dig. Dis. Sci..

[162] Coulson-Thomas V.J., Coulson-Thomas Y.M., Gesteira T.F., De Paula C.A.A., Mader A.M., Waisberg J., Pinhal M.A., Friedl A., Toma L., Nader H.B. (2011). Colorectal cancer desmoplastic reaction up-regulates collagen synthesis and restricts cancer cell invasion. Cell Tissue Res..

[163] Baker A.M., Bird D., Welti J.C., Gourlaouen M., Lang G., Murray G.I., Reynolds A.R., Cox T.R., Erler J.T. (2013). Lysyl oxidase plays a critical role in endothelial cell stimulation to drive tumor angiogenesis. Cancer Res..

[164] Kuczek D.E., Larsen A.M.H., Carretta M., Kalvisa A., Siersbæk M.S., Simões A.M.C., Roslindn A., Engelholm L.H., Donia M., Svane I.M. (2018). Collagen density regulates the activity of tumor-infiltrating T cells. bioRxiv.

[165] Wang E., Shibutani M., Nagahara H., Fukuoka T., Iseki Y., Okazaki Y., Kashiwagi S., Tanaka H., Maeda K., Hirakawa K. (2021). Abundant intratumoral fibrosis prevents lymphocyte infiltration into peritoneal metastases of colorectal cancer. PLoS ONE.

[166] Gordon-Weeks A., Lim Y., Yuzhalin E., Jones K., Markelc B., Buzelli N., Fokas E., Cao Y., Smart S., Muschel R. (2017). Neutrophils Promote Hepatic Metastasis Growth Through fibroblast growth factor (FGF)2-dependent Angiogenesis. Hepatology.

[167] Larsen M., Artym V.V., Green J.A., Yamada K.M. (2006). The matrix reorganized: Extracellular matrix remodeling and integrin signaling. Curr. Opin. Cell Biol..

[168] Liang Y., Lv Z., Huang G., Qin J., Li H., Nong F., Wen B. (2020). Prognostic significance of abnormal matrix collagen remodeling in colorectal cancer based on histologic and bioinformatics analysis. Oncol. Rep..

[169] Fagerholm S.C., Guenther C., Asens M.L., Savinko T., Uotila L.M. (2019). Beta2-Integins and interacting proteins in leukocyte trafficking, immune supression, and immunodeficiency disease. Front. Immunol..

[170] Misra S., Hascall V.C., Markwald R.R., Ghatak S. (2015). Interactions between hyaluronan and its receptors (CD44, RHAMM) regulate the activities of inflammation and cancer. Front. Immunol..

[171] Tammi M.I., Oikari S., Pasonen-Seppänen S., Rilla K., Auvinen P., Tammi R.H. (2019). Activated hyaluronan metabolism in the tumor matrix—Causes and consequences. Matrix Biol..

[172] Zhang G., Lu R., Wu M., Liu Y., He Y., Xu J., Yang C., Du Y., Gao F. (2019). Colorectal cancer-associated ~ 6 kDa hyaluronan serves as a novel biomarker for cancer progression and metastasis. FEBS J..

[173] Spaderna S., Schmalhofer O., Hlubek F., Berx G., Eger A., Merkel S., Jung A., Kirchner T., Brabletz T. (2006). A Transient, EMT-Linked Loss of Basement Membranes Indicates Metastasis and Poor Survival in Colorectal Cancer. Gastroenterology.

[174] Chang J., Chaudhuri O. (2019). Beyond proteases: Basement membrane mechanics and cancer invasion. J. Cell Biol..

[175] Kai F.B., Drain A.P., Weaver V.M. (2019). The Extracellular Matrix Modulates the Metastatic Journey. Dev. Cell.

[176] Li L., Song J., Chuquisana O., Hannocks M.J., Loismann S., Vogl T., Roth J., Hallmann R., Sorokin L. (2020). Endothelial Basement Membrane Laminins as an Environmental Cue in Monocyte Differentiation to Macrophages. Front. Immunol..

[177] Haessler U., Pisano M., Wu M., Swartz M.A. (2011). Dendritic cell chemotaxis in 3D under defined chemokine gradients reveals differential response to ligands CCL21 and CCL19. Proc. Natl. Acad. Sci. USA.

[178] Proudfoot A.E.I., Uguccioni M. (2016). Modulation of chemokine responses: Synergy and cooperativity. Front. Immunol..

[179] Franitza S., Hershkoviz R., Kam N., Lichtenstein N., Vaday G.G., Alon R., Lider O. (2000). TNF-α Associated with Extracellular Matrix Fibronectin Provides a Stop Signal for Chemotactically Migrating T Cells. J. Immunol..

[180] Martino M.M., Mochizuki M., Rothenfluh D.A., Rempel S.A., Hubbell J.A., Barker T.H. (2009). Controlling integrin specificity and stem cell differentiation in 2D and 3D environments through regulation of fibronectin domain stability. Biomaterials.

[181] Villalba M., Evans S.R., Vidal-Vanaclocha F., Calvo A. (2017). Role of TGF-β in metastatic colon cancer: It is finally time for targeted therapy. Cell Tissue Res..

[182] Wells R.G., Discher D.E. (2008). Matrix elasticity, cytoskeletal tension, and TGF-β: The insoluble and soluble meet. Sci. Signal..

[183] Buscemi L., Ramonet D., Klingberg F., Formey A., Smith-Clerc J., Meister J.J., Hinz B. (2011). The single-molecule mechanics of the latent TGF-β1 complex. Curr. Biol..

[184] Nishimura S.L. (2009). Integrin-mediated transforming growth factor-βactivation, a potential therapeutic target in fibrogenic disorders. Am. J. Pathol..

[185] Jha S.K., Rauniyar K., Karpanen T., Leppänen V.M., Brouillard P., Vikkula M., Alitalo K., Jeltsch M. (2017). Efficient activation of the lymphangiogenic growth factor VEGF-C requires the C-terminal domain of VEGF-C and the N-terminal domain of CCBE1. Sci. Rep..

[186] Akagi K., Ikeda Y., Miyazaki M., Abe T., Kinoshita J., Maehara Y., Sugimachi K. (2000). Vascular endothelial growth factor-C (VEGF-C) expression in human colorectal cancer tissues. Br. J. Cancer.

[187] Güç E., Briquez P.S., Foretay D., Fankhauser M.A., Hubbell J.A., Kilarski W.W., Swartz M.A. (2017). Local induction of lymphangiogenesis with engineered fibrin-binding VEGF-C promotes wound healing by increasing immune cell trafficking and matrix remodeling. Biomaterials.

[188] Fankhauser M., Broggi M.A.S., Potin L., Bordry N., Jeanbart L., Lund A.W., Da Costa E., Hauert S., Rincon-Restrepo M., Tremblay C. (2017). Tumor lymphangiogenesis promotes T cell infiltration and potentiates immunotherapy in melanoma. Sci. Transl. Med..

[189] Skobe M., Hamberg L.M., Hawighorst T., Schirner M., Wolf G.L., Alitalo K., Detmar M. (2001). Concurrent induction of lymphangiogenesis, angiogenesis, and macrophage recruitment by vascular endothelial growth factor-C in melanoma. Am. J. Pathol..

[190] Castello L.M., Raineri D., Salmi L., Clemente N., Vaschetto R., Quaglia M., Garzaro M., Gentilli S., Navalesi P., Cantaluppi V. (2017). Osteopontin at the Crossroads of Inflammation and Tumor Progression. Mediat. Inflamm..

[191] Klement J.D., Paschall A.V., Redd P.S., Ibrahim M.L., Lu C., Yang D., Celis E., Abrams S.I., Ozato K., Liu K. (2018). An osteopontin/CD44 immune checkpoint controls CD8+ T cell activation and tumor immune evasion. J. Clin. Invest..

[192] Shurin M.R. (2018). Osteopontin controls immunosuppression in the tumor microenvironment. J. Clin. Investig..

[193] Julier Z., Martino M.M., de Titta A., Jeanbart L., Hubbell J.A. (2015). The TLR4 agonist fibronectin extra domain a is cryptic, Exposed by elastase-2; Use in a fibrin matrix cancer vaccine. Sci. Rep..

[194] Hope C., Emmerich P.B., Papadas A., Pagenkopf A., Matkowskyj K.A., Van De Hey D.R., Payne S.N., Clipson L., Callander N.S., Hematti P. (2017). Versican-Derived Matrikines Regulate Batf3–Dendritic Cell Differentiation and Promote T Cell Infiltration in Colorectal Cancer. J. Immunol..

[195] Oldberg Å., Kalamajski S., Salnikov A.V., Stuhr L., Mörgelin M., Reed R.K., Heldin N.E., Rubin K. (2007). Collagen-binding proteoglycan fibromodulin can determine stroma matrix structure and fluid balance in experimental carcinoma. Proc. Natl. Acad. Sci. USA.

[196] Sjöberg P., Manderson A., Mögelin M., Heinegard D., Mlom M. (2010). Short leucine-rich glycoproteins of the extracellular matrix display diverse patterns of complement interaction and activation. Mol. Immunol..

[197] Jiménez-Gastélum G.R., Aguilar-Medina E.M., Soto-Sainz E., Ramos-Payán R., Silva-Benítez E.L. (2019). Antimicrobial Properties of Extracellular Matrix Scaffolds for Tissue Engineering. BioMed Res. Int..

[198] Alfano M., Canducci F., Nebuloni M., Clementi M., Montorsi F., Salonia A. (2016). The interplay of extracellular matrix and microbiome in urothelial bladder cancer. Nat. Rev. Urol..

[199] Villalba N., Baby S., Yuan S.Y. (2021). The Endothelial Glycocalyx as a Double-Edged Sword in Microvascular Homeostasis and Pathogenesis. Front. Cell Dev. Biol..

[200] Möckl L., Pedram K., Roy A.R., Krishnan V., Gustavsson A.K., Dorigo O., Bertozzi C.R., Moerner W.E. (2019). Quantitative Super-Resolution Microscopy of the Mammalian Glycocalyx. Dev. Cell.

[201] Paszek M.J., Dufort C.C., Rossier O., Bainer R., Mouw J.K., Godula K., Hudak J.E., Lakins J.N., Wijekoon A.C., Cassereau L. (2014). The cancer glycocalyx mechanically primes integrin-mediated growth and survival. Nature.

[202] Kanyo N., Kovacs K.D., Saftics A., Szekacs I., Peter B., Santa-Maria A.R., Walter F.R., Dér A., Deli M.A., Horvath R. (2020). Glycocalyx regulates the strength and kinetics of cancer cell adhesion revealed by biophysical models based on high resolution label-free optical data. Sci. Rep..

[203] Ghasempour S., Freeman S.A. (2021). The glycocalyx and immune evasion in cancer. FEBS J..

[204] Hu Z., Cano I., D’Amore P.A. (2021). Update on the Role of the Endothelial Glycocalyx in Angiogenesis and Vascular Inflammation. Front. Cell Dev. Biol..

[205] Mensah S.A., Harding I.C., Zhang M., Jaeggli M.P., Torchilin V.P., Niedre M.J., Ebong E.E. (2019). Metastatic cancer cell attachment to endothelium is promoted by endothelial glycocalyx sialic acid degradation. AIChE J..

[206] Mensah S.A., Nersesyan A.A., Harding I.C., Lee C.I., Tan X., Banerjee S., Niedre M., Torchilin V.P., Ebong E.E. (2020). Flow-regulated endothelial glycocalyx determines metastatic cancer cell activity. FASEB J..

[207] Hanahan D. (2022). Hallmarks of Cancer: New Dimensions. Cancer Discov..

[208] Nejman D., Livyatan I., Fuks G., Gavert N., Zwnag Y., Geller T., Rotter-Maskowitz A., Weiser R., Mallel G., Gigi E. (2020). The human tumor microbiome is composed of tumor type-specific intracellular bacteria. Science.

[209] Brody H. (2020). The gut microbiome. Nature.

[210] Thorbeck G.J. (1959). Some Histological and Functional Aspects of Lymphoid Tissue in Germ Free Animals: I Morphological Studies. Ann. N. Y. Acad. Sci..

[211] Hill A., Artis D. (2010). Intestinal Bacteria and the Regulation of Immune Cell Homeostasis. Annu. Rev. Immunol.

[212] Shi N., Li N., Duan X., Niu H. (2017). Interaction between the gut microbiome and mucosal immune system. Mil. Med. Res..

[213] Round J.L., Mazmanian S.K. (2009). The gut microbiota shapes intestinal immune responses during health and disease. Nat. Rev. Immunol..

[214] Zheng D., Liwinski T., Elinav E. (2020). Interaction between microbiota and immunity in health and disease. Cell Res..

[215] Mishima Y., Oka A., Liu B., Herzog J.W., Eun C.S., Fan T.J., Bulik-Sullivan E., Carroll I.M., Hansen J.J., Chen L. (2019). Microbiota maintain colonic homeostasis by activating TLR2/MyD88/PI3K signaling in IL-10-producing regulatory B cells. J. Clin. Invest..

[216] Atarashi K., Tanoue T., Oshima K., Suda W., Nagano Y., Nishikawa H., Fukuda S., Saito T., Narushima S., Hase K. (2013). Treg induction by a rationally selected mixture of Clostridia strains from the human microbiota. Nature.

[217] Kayama H., Takeda K. (2014). Polysaccharide A of bacteroides fragilis: Actions on dendritic cells and T cells. Mol. Cell.

[218] Guo M., Xu E., Ai D. (2019). Inferring bacterial infiltration in primary colorectal tumors from host whole genome sequencing data. Front. Genet..

[219] Abed J., Emgård J.E.M., Zamir G., Faroja M., Almogy G., Grenov A., Sol A., Naor R., Pikarsky E., Atlan K.A. (2016). Fap2 Mediates Fusobacterium nucleatum Colorectal Adenocarcinoma Enrichment by Binding to Tumor-Expressed Gal-GalNAc. Cell Host Microbe.

[220] Kaplan C.W., Ma X., Paranjpe A., Jewett A., Lux R., Kinder-Haake S., Shi W. (2010). Fusobacterium nucleatum outer membrane proteins Fap2 and RadD induce cell death in human lymphocytes. Infect. Immun..

[221] Park H.E., Kim J.H., Cho N.Y., Lee H.S., Kang G.H. (2017). Intratumoral Fusobacterium nucleatum abundance correlates with macrophage infiltration and CDKN2A methylation in microsatellite-unstable colorectal carcinoma. Virchows Arch..

[222] Hamada T., Zhang X., Mima K., Bullman S., Sukawa Y., Nowak J.A., Kosumi K., Masugi Y., Twombly T.S., Cao Y. (2018). Fusobacterium nucleatum in colorectal cancer relates to immune response differentially by tumor microsatellite instability status. Cancer Immunol. Res..

[223] Li S., Simoni Y., Becht E., Loh C.Y., Li N., Lachance D., Koo S.L., Lim T.P., Tan E.K.W., Mathew R. (2020). Human Tumor-Infiltrating MAIT Cells Display Hallmarks of Bacterial Antigen Recognition in Colorectal Cancer. Cell Rep. Med..

[224] Cremonesi E., Governa V., Garzon J.F.G., Mele V., Amicarella F., Muraro M.G., Trella E., Galati-Fournier V., Oertli D., Däster S.R. (2018). Gut microbiota modulate T cell trafficking into human colorectal cancer. Gut.

[225] Roberti M.P., Yonekura S., Duong C.P.M., Picard M., Ferrere G., Tidjani Alou M., Rauber C., Iebba V., Lehmann C.H.K., Amon L. (2020). Chemotherapy-induced ileal crypt apoptosis and the ileal microbiome shape immunosurveillance and prognosis of proximal colon cancer. Nat. Med..

[226] Toussi D.N., Liu X., Massari P. (2012). The FomA porin from Fusobacterium nucleatum is a toll-like receptor 2 agonist with immune adjuvant activity. Clin. Vaccine Immunol..

[227] Park S.R., Kim D.J., Han S.H., Kang M.J., Lee J.Y., Jeong Y.J., Lee S.J., Kim T.H., Ahn S.G., Yoon J.H. (2014). Diverse toll-like receptors mediate cytokine production by fusobacterium nucleatum and aggregatibacter actinomycetemcomitans in macrophages. Infect. Immun..

[228] Maisonneuve C., Tsang D.K.L., Foerster E.G., Robert L.M., Mukherjee T., Prescott D., Tattoli I., Lemire P., Winer D.A., Winer S. (2021). Nod1 promotes colorectal carcinogenesis by regulating the immunosuppressive functions of tumor-infiltrating myeloid cells. Cell Rep..

[229] Lamprinaki D., Garcia-Vello P., Marchetti R., Hellmich C., McCord K.A., Bowles K.M., Macauley M.S., Silipo A., De Castro C., Crocker P.R. (2021). Siglec-7 Mediates Immunomodulation by Colorectal Cancer-Associated Fusobacterium nucleatum ssp. animalis. Front. Immunol..

[230] Martin-Gallausiaux C., Malabirade A., Habier J., Wilmes P. (2020). Fusobacterium nucleatum Extracellular Vesicles Modulate Gut Epithelial Cell Innate Immunity via FomA and TLR2. Front. Immunol..

[231] Fung K.Y.C., Cosgrove L., Lockett T., Head R., Topping D.L. (2012). A review of the potential mechanisms for the lowering of colorectal oncogenesis by butyrate. Br. J. Nutr..

[232] Okumura S., Konishi Y., Narukawa M., Sugiura Y., Yoshimoto S., Arai Y., Sato S., Yoshida Y., Tsuji S., Uemura K. (2021). Gut bacteria identified in colorectal cancer patients promote tumourigenesis via butyrate secretion. Nat. Commun..

[233] Nakkarach A., Foo H.L., Song A.A.L., Mutalib N.E.A., Nitisinprasert S., Withayagiat U. (2021). Anti-cancer and anti-inflammatory effects elicited by short chain fatty acids produced by Escherichia coli isolated from healthy human gut microbiota. Microb. Cell Fact..

[234] Furusawa Y., Obata Y., Fukuda S., Endo T.A., Nakato G., Takahashi D., Nakanishi Y., Uetake C., Kato K., Kato T. (2013). Commensal microbe-derived butyrate induces the differentiation of colonic regulatory T cells. Nature.

[235] He Y., Fu L., Li Y., Wang W., Gong M., Zhang J., Dong X., Huang J., Wang Q., Mackay C.R. (2021). Gut microbial metabolites facilitate anticancer therapy efficacy by modulating cytotoxic CD8+ T cell immunity. Cell Metab..

[236] Silva Y.P., Bernardi A., Frozza R.L. (2020). The Role of Short-Chain Fatty Acids From Gut Microbiota in Gut-Brain Communication. Front. Endocrinol..

[237] Boleij A., Hechenbleikner E.M., Goodwin A.C., Badani R., Stein E.M., Lazarev M.G., Ellis B., Carroll K.C., Albesiano E., Wick E.C. (2015). The bacteroides fragilis toxin gene is prevalent in the colon mucosa of colorectal cancer patients. Clin. Infect. Dis..

[238] Wilson M.R., Jiang Y., Villalta P.W., Stornetta A., Boudreau P.D., Carrá A., Brennan C.A., Chun E., Ngo L., Samson L.D. (2019). The human gut bacterial genotoxin colibactin alkylates DNA. Science.

[239] Wu S., Rhee K.J., Albesiano E., Rabizadeh S., Wu X., Yen H.R., Huso D.L., Brancati F.L., Wick E., McAllister F. (2009). A human colonic commensal promotes colon tumorigenesis via activation of T helper type 17 T cell responses. Nat. Med..

[240] Dalmasso G., Cougnoux A., Delmas J., Darfeuille-Michaud A., Bonnet R. (2015). The bacterial genotoxin colibactin promotes colon tumor growth by modifying the tumor microenvironment. Gut Microbes.

[241] Proença M.A., Biselli J.M., Succi M., Severino F.E., Berardinelli G.N., Caetano A., Reis R.M., Hughes D.J., Silva A.E. (2018). Relationship between fusobacterium nucleatum, inflammatory mediators and microRNAs in colorectal carcinogenesis. World J. Gastroenterol..

[242] Prorok-Hamon M., Friswell M.K., Alswied A., Roberts C.L., Song F., Flanagan P.K., Knight P., Codling C., Marchesi J.R., Winstanley C. (2014). Colonic mucosa-associated diffusely adherent afaC+ Escherichia coli expressing lpfA and pks are increased in inflammatory bowel disease and colon cancer. Gut.

